# Supplementary material for: RXRs control serous macrophage neonatal expansion and identity and contribute to ovarian cancer progression
Source: Nat Commun. 2020 Apr 3;11:1655. doi: 10.1038/s41467-020-15371-0 (PMC7125161; doi:10.1038/s41467-020-15371-0)
Supplement: Supplementary file 1 — Supplementary Information [file 41467_2020_15371_MOESM1_ESM.pdf]

Supplementary information

RXRs control serous macrophage neonatal expansion and identity and contribute to ovarian cancer progression

María Casanova-Acebes, María Piedad Menéndez-Gutiérrez, Jesús Porcuna, Damiana Álvarez-Errico, Yonit Lavin, Ana García, Soma Kobayashi, Jessica Le Berichel, Vanessa Núñez, Felipe Were, Daniel Jiménez-Carretero, Fátima Sánchez-Cabo, Miriam Merad, Mercedes Ricote

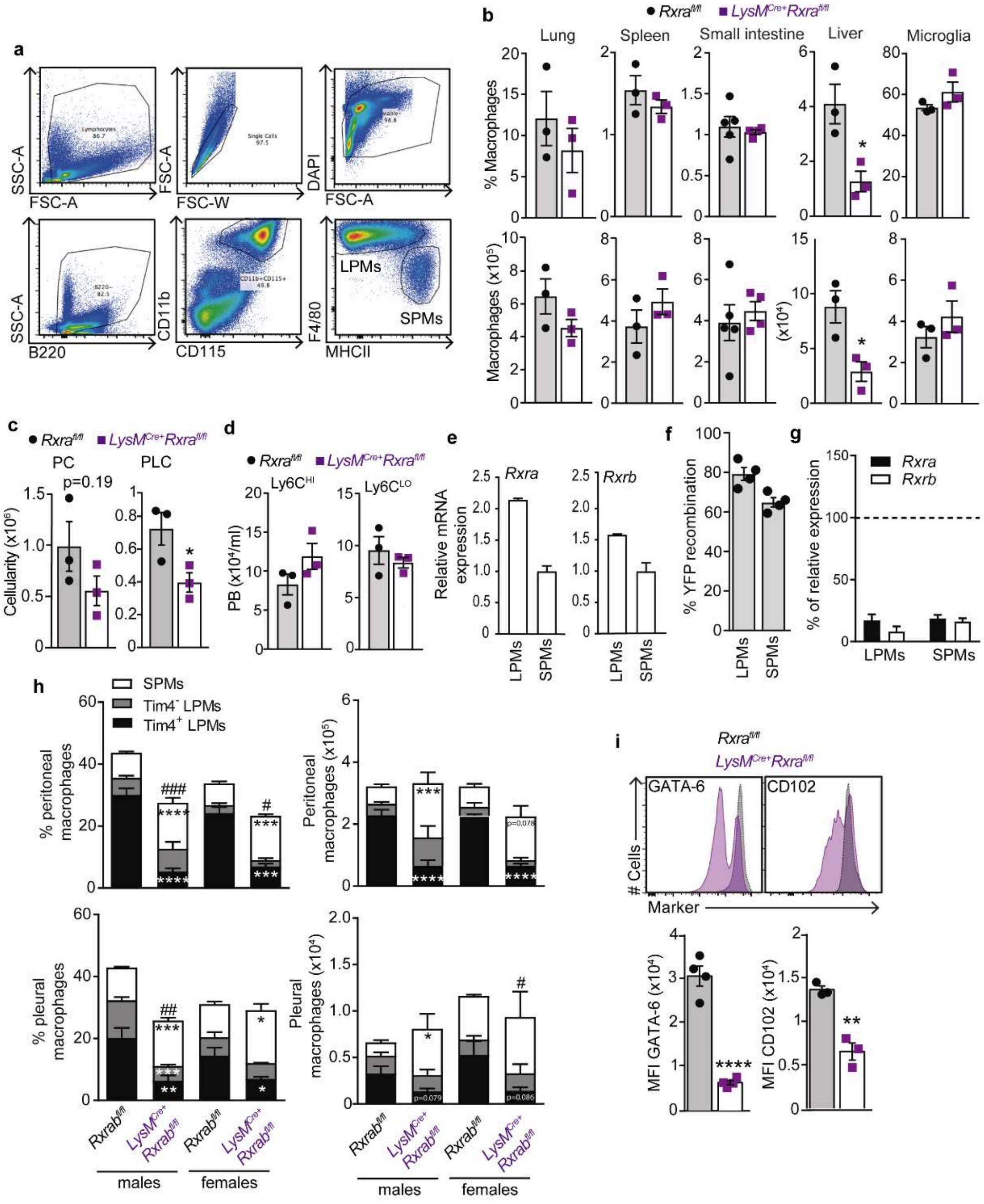

Supplementary Fig. 1 LPM gating strategy and characterization of *LysM*$^{Cre+}$*Rxra*$^{fl/fl}$ mice. **a**, Gating strategy used to identify LPMs and SPMs in serous cavities. **b**, Frequencies and absolute quantification for TRMs in lung, spleen, small intestine, liver and microglia defined as in Lavin et al., 2014[1]. N=3, representative of two to three independent experiments. **c**, Quantification of peritoneal and pleural cellularity in *LysM*$^{Cre+}$*Rxra*$^{fl/fl}$ and *Rxra*$^{fl/fl}$ mice. N=3 mice, representative of two independent experiments. PC: peritoneal cavity; PLC: pleural cavity. **d**, Absolute number of circulating Ly6C^{HI} and Ly6C^{LO} monocytes defined as CD45^{+}Ly6G^{NEG}CD11b^{+}CD115^{+} by flow cytometry in *LysM*$^{Cre+}$*Rxra*$^{fl/fl}$ and

Rxra$^{fl/fl}$ mice. N=3 per genotype, representative of two independent experiments. **e**, Relative mRNA expression of *Rxra* and *Rxrb* isoforms in peritoneal LPMs and SPMs. N=3. **f**, Percentage of YFP+ cells in peritoneal LPMs and SPMs, using *LysM*$^{Cre+}$R26-YFP mice. N=4. **g**, Percentage of mRNA expression of *Rxra* and *Rxrb* in *LysM*$^{Cre+}$*Rxra*b$^{fl/fl}$ mice compared with *Rxrab*$^{fl/fl}$ littermate controls (dashed line), determined by qPCR using primers from regions upstream and downstream the deleted exons. N=10-11. **h**, Flow cytometry of serous cavities from 9 week-old *LysM*$^{Cre+}$*Rxrab*$^{fl/fl}$ mice and *Rxrab*$^{fl/fl}$ littermates separated by gender (see also Fig. 1d-e). Graphs show frequencies among CD45^{+} leukocytes and absolute numbers. Data (n=3-11 per genotype and gender) are pooled from two independent experiments; #p≤0.05; ##p≤0.01; ###p≤0.001 (unpaired Student's t test) *versus* total macrophage percentage or absolute numbers in *Rxrab*$^{fl/fl}$ mice; *p≤0.05; **p≤0.01; ***p≤0.001; ****p≤0.0001 (unpaired Student's t test) versus the same population in *Rxrab*$^{fl/fl}$ mice. **i**, MFI (mean fluorescent intensity) for GATA-6 and CD102 markers determined by flow cytometry in peritoneal LPMs of *LysM*$^{Cre+}$*Rxra*$^{fl/fl}$ (purple) and *Rxra*$^{fl/fl}$ (gray) mice. N=3-4 per genotype, representative of three independent experiments. All data are represented as mean±SEM; *p≤0.05; **p≤0.01; ***p≤0.01 (unpaired Student's t test). Source data are provided as a Source Data file.

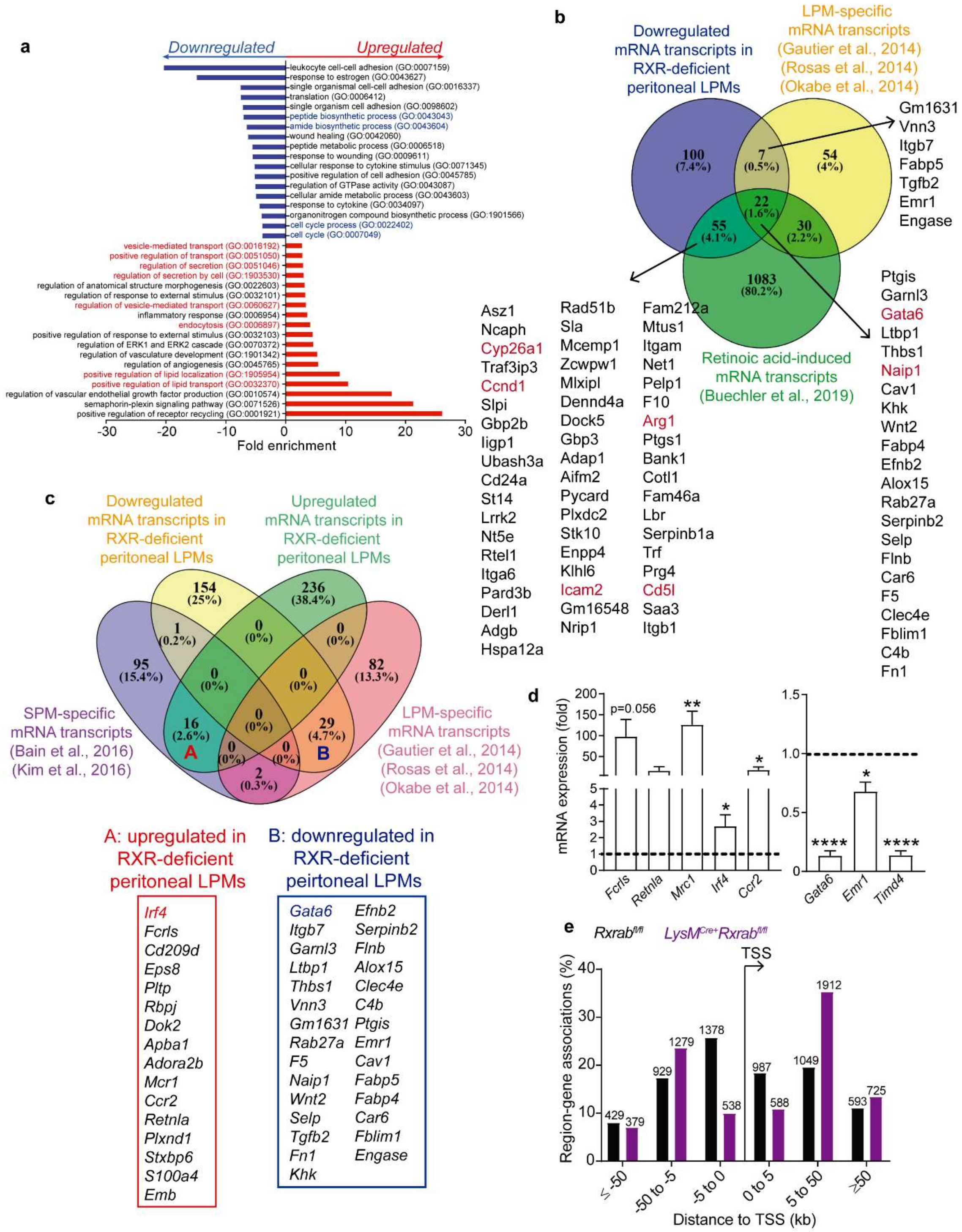

Supplementary Fig. 2 RXRs control LPM signature. **a**, List of significantly enriched GO terms (fold enrichment levels ≥ 2.5 and ≤ -4) in *LysM*$^{Cre+}$*Rxra*$^{fl/fl}$ versus *Rxra*$^{fl/fl}$ LPMs for diverse biological processes and categories upon analysis of the RNA-seq data set with the PANTHER statistical over-representation test tool. p≤0.05. **b**, Venn diagram (Venny 2.1.0) of overlapping groups of downregulated genes in

LysM^{Cre+}*Rxra*$^{fl/fl}$ *versus Rxra*$^{fl/fl}$ LPMs from our RNA-seq data set (blue), as well as previously published retinoic acid signalin-dependent[2] (green), and LPM-specific (yellow; downregulated in GATA-6-deficient LPMs)[3, 4, 5] genes. Number and relative frequencies of gene RNA-seq profiling are displayed per signature intersection. **c**, Venn diagram (Venny 2.1.0) of overlapping groups of upregulated (green) and downregulated genes (yellow) in *LysM*$^{Cre+}$*Rxra*$^{fl/fl}$ *versus Rxra*$^{fl/fl}$ LPMs from our RNA-seq data set, as well as previously published SPM-specific[6, 7] (purple) and LPM-specific[3, 4, 5] (pink; downregulated in GATA-6-deficient LPMs) genes. The lower red square (A) lists peritoneal SPM genes upregulated in *LysM*$^{Cre+}$*Rxra*$^{fl/fl}$ peritoneal LPMs, whereas the blue box (B) represents genes commonly downregulated in GATA-6-deficient and *LysM*$^{Cre+}$*Rxra*$^{fl/fl}$ peritoneal LPMs. Number and relative frequencies of gene RNA-seq profiling are displayed per signature intersection. **d**, Relative mRNA expression for SPMs (left) and LPMs (right) genes in adult peritoneal LPMs of *LysM*$^{Cre+}$*Rxrab*$^{fl/fl}$ mice determined by qPCR. Gene expression is normalized to *Rxrab*$^{fl/fl}$ peritoneal LPMs (dashed line). N=3-5 per genotype and gene. **e**, Distribution of genomic features of differential regulatory elements by distance to the TSS, assessed by ATAC-seq analysis of *Rxrab*$^{fl/fl}$ and *LysM*$^{Cre+}$*Rxrab*$^{fl/fl}$ peritoneal LPMs. Graph shows the percentage, and numbers on top of bars indicate total number of peaks in each bounded distance. All data are represented as mean±SEM. *p≤0.05; **p≤0.01; ***p≤0.001 (unpaired Student's t test). Source data are provided as a Source Data file.

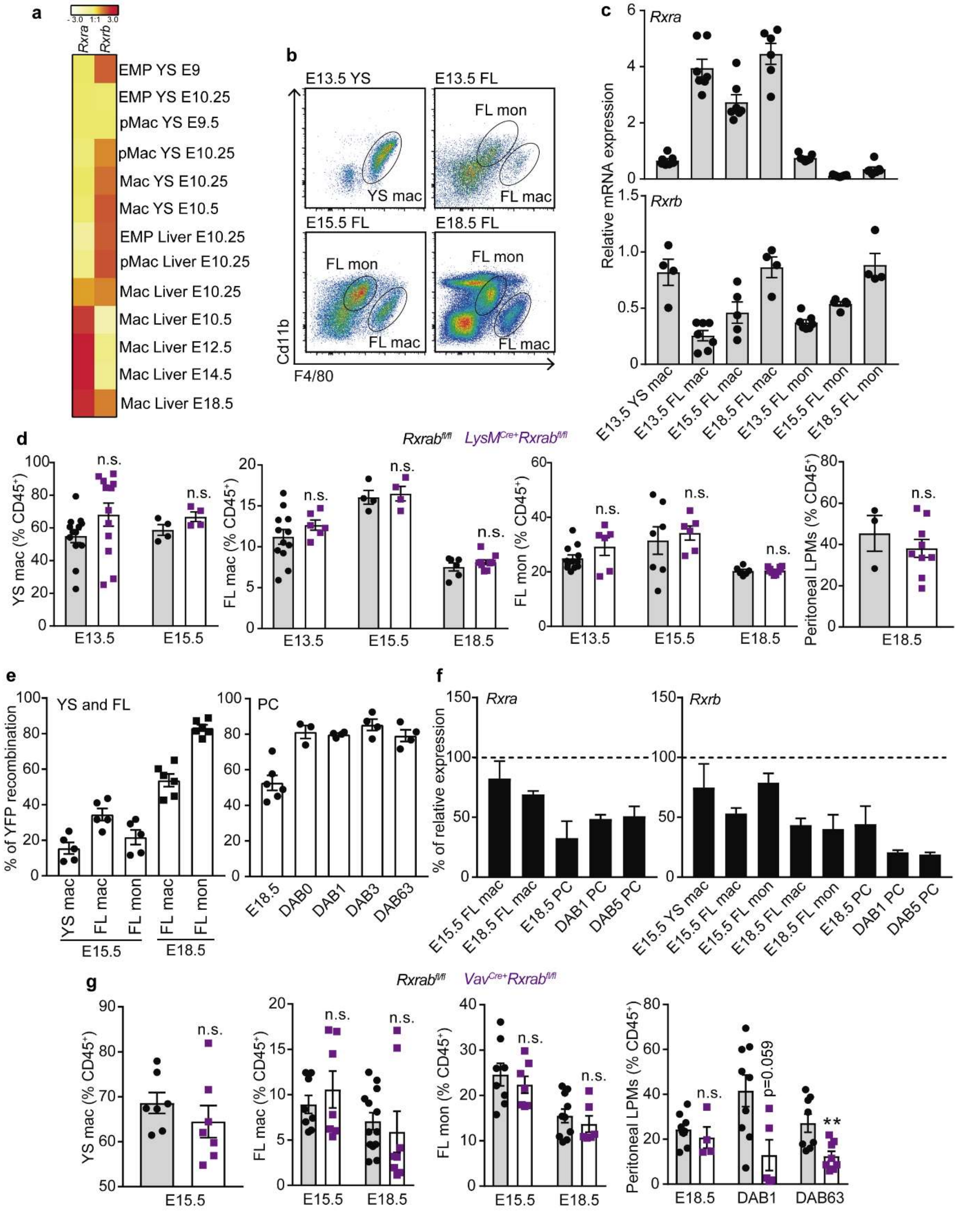

Supplementary Fig. 3 RXRs are dispensable for the embryonic development of LPMs. **a**, Expression of mRNA for *Rxra* and *Rxr*b throughout embryonic macrophage precursors from RNA-sequencing data published by Mass et al., 2016[8]. Heatmap shows normalized reads. Data are displayed from white (downregulated) to red (upregulated), as shown in the colour gradient (log2 normalized gene expression). **b**, Representative flow cytometry dot plots of E13.5 yolk sac macrophages (Singlets

CD45$^+$CD11b$^+$Ly6C$^-$F4/80^{HI}) as well as E13.5, E15.5 and E18.5 fetal liver macrophages (Singlets CD45$^+$CD11b$^+$Ly6C$^-$F4/80^{HI}) and monocytes (Singlets CD45$^+$CD11b$^+$Ly6C$^{+/-}$F4/80^{INT}) in embryos from *Rxrab*$^{fl/fl}$ mice. **c**, Relative mRNA expression of *Rxra* and *Rxrb* in embryonic macrophages and monocytes sorted as shown in (**b**); N=4-7 from up to two litters per age. **d**, Flow cytometry of yolk sac macrophages and fetal liver macrophages and monocytes as defined in (**b**), as well as E18.5 peritoneal LPMs (CD11b$^+$F4/80^{HI}MHCII^{LO}TIM4$^+$) from *LysM*$^{Cre+}$*Rxrab*$^{fl/fl}$ and *Rxrab*$^{fl/fl}$ mice. Graphs show the frequency of cells among CD45$^+$ leukocytes in E13.5, E15.5 and E18.5 embryos. N=3-13, pooled from up to three litters per age. **e**, Percentage of YFP$^+$ recombination in embryonic macrophages using LysM^{Cre+}R26-LSL-YFP mice. N=3-6 per cell type, pooled from up to two litters per age. **f**, Percentage of mRNA expression of *Rxra* and *Rxrb* in *LysM*$^{Cre+}$*Rxrab*$^{fl/fl}$ cells compared with cells from age-paired *Rxrab*$^{fl/fl}$ littermate controls (dashed line), determined by qPCR using primers from regions upstream and downstream the deleted exons[9]. N=3-13 per age, pooled from up to two independent experiments per age. **g**, Flow cytometry of yolk sac macrophages and fetal liver macrophages and monocytes as defined in (**b**), as well as E18.5, DAB1 and DAB63 peritoneal LPMs (CD11b$^+$F4/80^{HI}MHCII^{LO}TIM4$^+$) from *Vav*$^{Cre+}$*Rxrab*$^{fl/fl}$ and *Rxrab*$^{fl/fl}$ mice. Graphs show the frequency of cells among CD45$^+$ leukocytes in E15.5 and E18.5 embryos, as well as 1- or 63-day-old mice. N=4-10, pooled from up to two litters per age. All data are presented as mean±SEM. n.s., no significant (unpaired Student's t test); EMP, erythroid myeloid progenitors; pMac, pre-macrophages; Mac, macrophages; YS, yolk sac; PC, peritoneal cavity; DAB, day after birth. Source data are provided as a Source Data file.

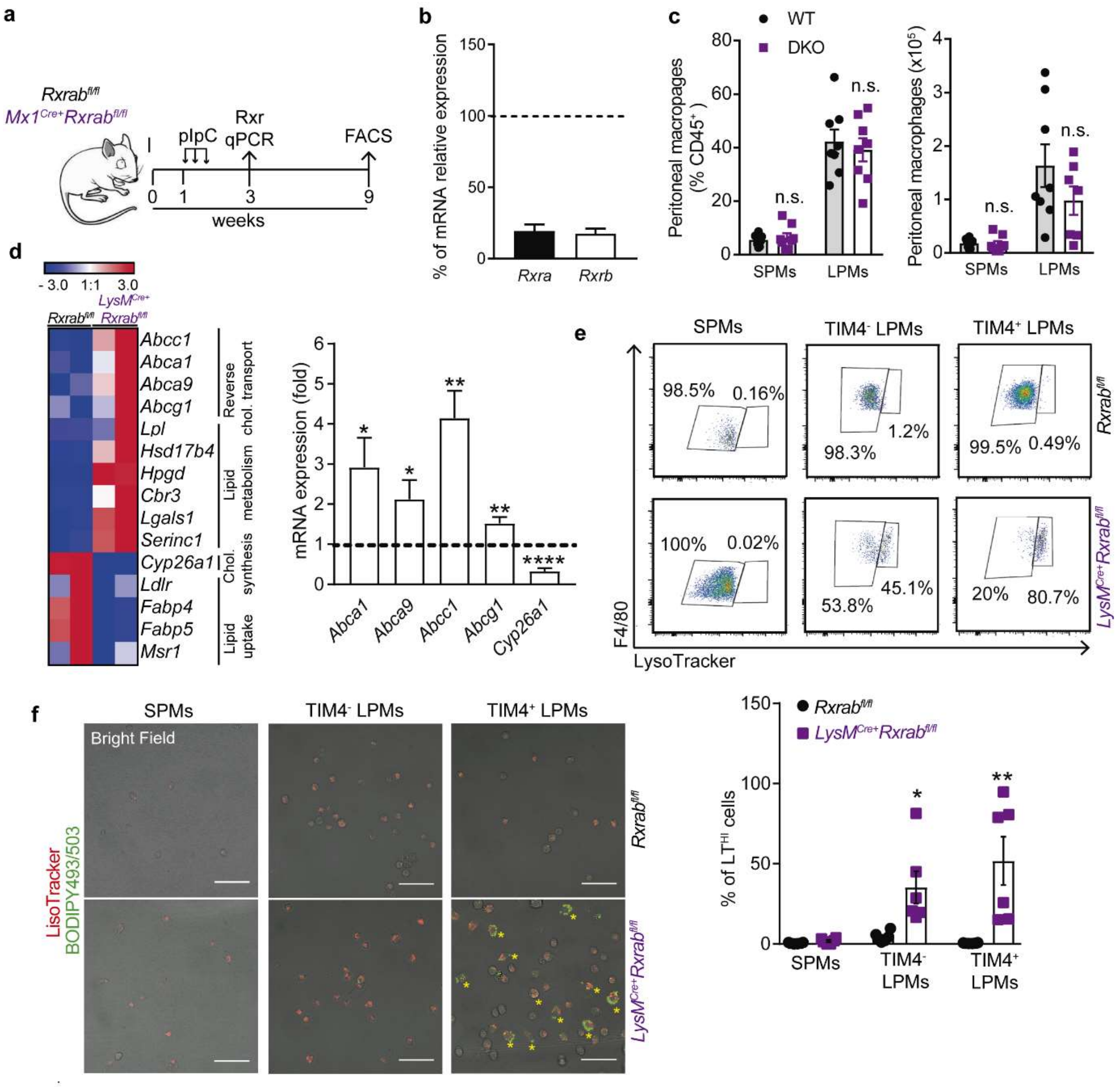

Supplementary Fig. 4 RXR deficiency leads to lipid accumulation and apoptosis of TIM4^{+} LPMs. **a-c**, Analysis of the peritoneal macrophage content of *Mx1*$^{\text{Cre+}}$*Rxrab*$^{\text{fl/fl}}$ and *Rxrab*$^{\text{fl/fl}}$ mice; **a**, Experimental desing; **b**, Percentage of mRNA expression of *Rxra* and *Rxrb* in *Mx1*$^{\text{Cre+}}$*Rxrab*$^{\text{fl/fl}}$ mice compared with *Rxrab*$^{\text{fl/fl}}$ LPMs (dashed line), determined by qPCR using primers from regions upstream and downstream the deleted exons. N=6; **c**, Flow cytometry of the peritoneal cavity from *Mx1*$^{\text{Cre+}}$*Rxrab*$^{\text{fl/fl}}$ mice and *Rxrab*$^{\text{fl/fl}}$ littermates, showing frequencies among CD45^{+} leukocytes and absolute numbers. N=7-8, pooled from two independent experiments. **d**, Heatmap showing normalized log2 FC in the expression of lipid homeostasis-related genes in adult *LysM*$^{\text{Cre+}}$*Rxra*$^{\text{fl/fl}}$ and *Rxra*$^{\text{fl/fl}}$ LPMs (left), and qPCR analysis of *LysM*$^{\text{Cre+}}$*Rxrab*$^{\text{fl/fl}}$ and *Rxrab*$^{\text{fl/fl}}$ LPMs (right). N=4-6 per genotype and gene. **e**, LysoTracker flow cytometry plots (up) and frequency (down) of LysoTracker^{HI} peritoneal SPMs, TIM4^{-}

LPMs and TIM4$^+$ LPMs in *LysM*$^{Cre+}$*Rxrab*$^{fl/fl}$ mice and *Rxrab*$^{fl/fl}$ littermates. N=4-6 per genotype, pooled from three independent experiments. **f**, Confocal images showing overlaid channels for LysoTracker (red), BODIPY493/503 (green) and brightfield from sorted and cultured macrophages from the peritoneal cavity of *LysM*$^{Cre+}$*Rxrab*$^{fl/fl}$ and control *Rxrab*$^{fl/fl}$ mice. Asterisks mark LysoTracker^{HI} BODIPY493/503^{HI} cells. N=6. Scale bar=100μm. All data are presented as mean±SEM. n.s. (no significant); *p≤0.05; **p≤0.01 (unpaired Student's t test). Source data are provided as a Source Data file.

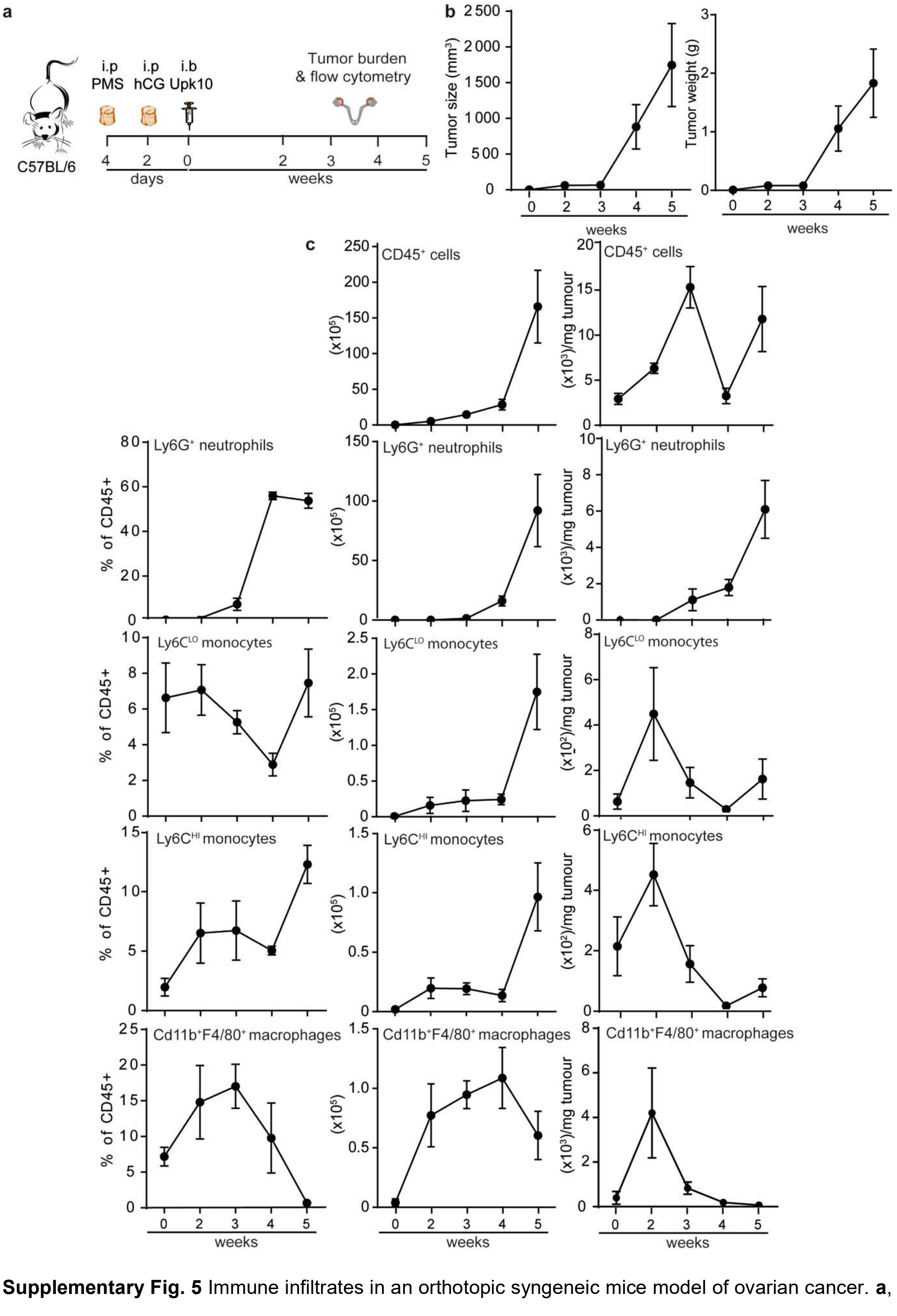

Supplementary Fig. 5 Immune infiltrates in an orthotopic syngeneic mice model of ovarian cancer. **a**, Experimental design of the orthotopic model with the Upk10 ovarian tumour cell line. i.p., intraperitoneal;

i.b., intrabursal; PMS, pregnant mare serum gonadotropin; hCG, human chorionic gonadotropin; Upk10, mouse ovarian tumour cell line. **b**, Primary ovarian tumour growth in volume (mm^3) and weight measured weekly in C57BL/6 wild type mice. N=4-5 per time point. **c**, Flow cytometry analysis of leukocytes isolated from primary ovarian tumours. Graphs represent frequencies among $CD45^+$ leukocytes, absolute numbers, and absolute numbers/mg of tumour, of cells pre-gated on singlets/$DAPI^{NEG}$/$Upk10^-$/$B220^-$CD3- cells. N=3-4 per time point. Data are represented as mean±SEM. Source data are provided as a Source Data file.

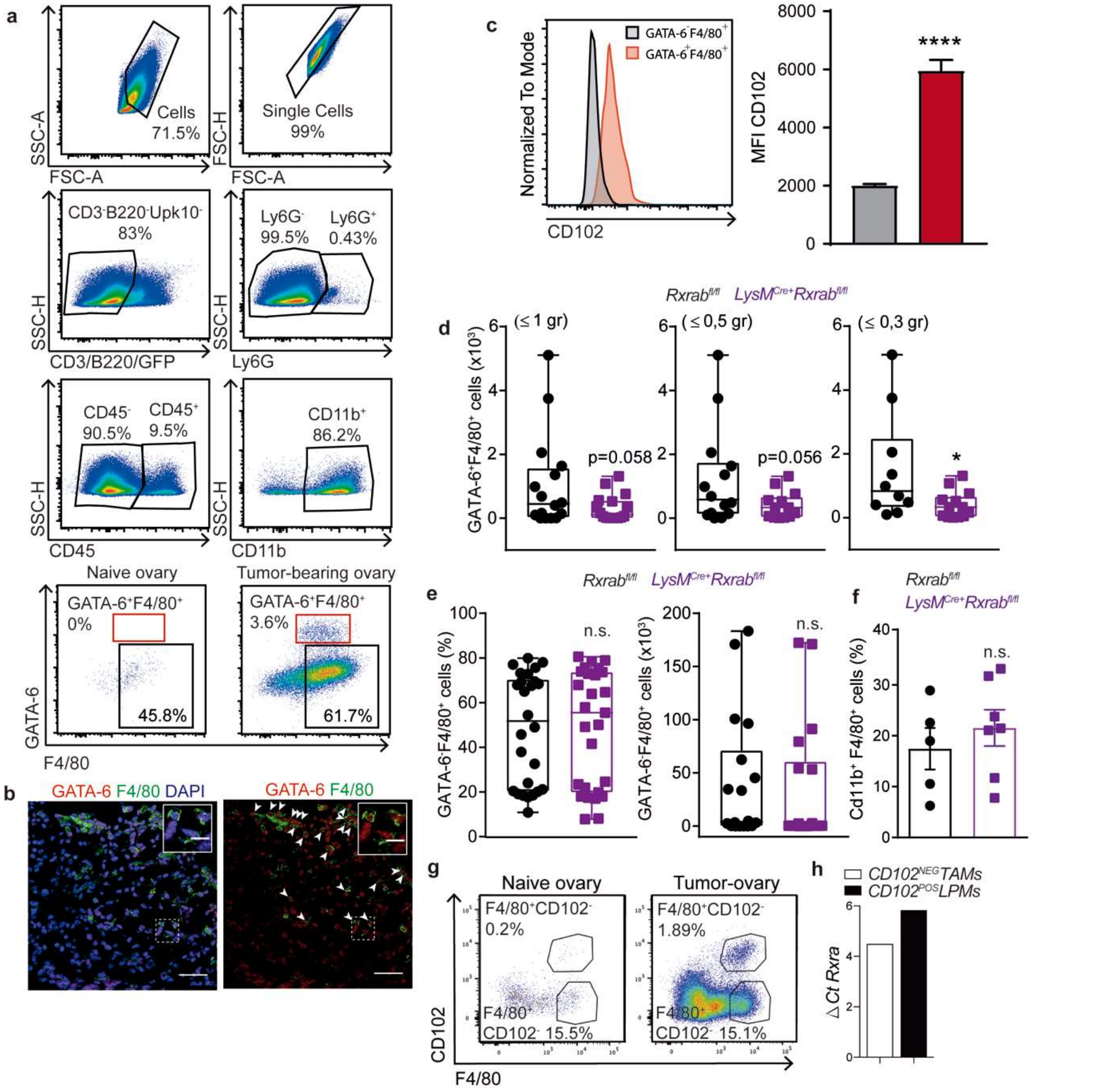

Supplementary Fig. 6 Peritoneal cavity macrophages contribute to ovarian tumour progression. **a**, Gating strategy used to identify F4/80$^+$GATA-6$^+$ macrophages within primary ovarian tumours from Upk10-injected C57BL/6 wild type mice. Frequency of F4/80$^+$GATA-6$^+$ cells in naïve and tumour-bearing ovaries are shown. **b**, Representative immunofluorescence staining for GATA-6 (red), F4/80 (green) and DAPI (blue) of tumour sections. Scale bars: 100 μm in full images, and 10 μm in cropped images. **c**, Flow cytometry histograms and quantification for mean fluorescence intensity (MFI) of CD102 in F4/80$^+$GATA-6$^+$ and F4/80$^+$GATA-6$^-$ cells within primary ovarian tumours from Upk10 injected C57BL76 wild type mice. N=5 (unpaired Student's t test). **d**, GATA-6$^+$F4/80$^+$ cells pre-gated on CD45$^+$CD11b$^+$ cells in *LysM*$^{\mathrm{Cre+}}$*Rxrab*$^{\mathrm{fl/fl}}$ and *Rxrab*$^{\mathrm{fl/fl}}$ mice, 24 days post-Upk10 injection. Data are stratified by tumour weight (0.3-1g). N=10-16, pooled from four independent experiments. Statistics

show generalized linear model with gamma distribution. **e**, Relative quantification and absolute numbers of GATA-6^{-}F4/80^{+} cells pre-gated on CD45^{+}CD11b^{+} cells in *LysM*$^{Cre+}$*Rxrab*$^{fl/fl}$ and *Rxrab*$^{fl/fl}$ mice, 24 days post-Upk10 injection. N=18-27 per genotype, pooled from four independent experiments. Statistics show generalized linear model with gamma distribution for absolute numbers, and Mann Whitney u test for frequency. **f**, Graph showing the frequency (% of CD45^{+} cells) of CD11b^{+}F4/80^{+} ovarian resident macrophages (pre-gated on CD45^{+}B220^{-} cells) in 9 week-old *LysM*$^{Cre+}$*Rxrab*$^{fl/fl}$ and *Rxrab*$^{fl/fl}$ mice. N=5-6 per genotype, pooled from two independent experiments (unpaired Student's t test). **g**, Flow cytometry plots from naïve and Upk10 ovarian tumours analyzed at day 24 post-tumour injection. Cells were gated as singlets/DAPI^{NEG}B220^{NEG}CD3^{NEG}Ly6G^{NEG}F4/80^{+} and sorted based on CD102 expression. **h**, Quantitative PCR levels (represented as Delta Ct for absolute quantification) of *Rxra* transcripts for F4/80^{+}CD102^{NEG} TAMs and F4/80^{+}CD102^{+} LPMs. Data are represented as mean±SEM (**c** and **f**), or as median (lower-upper quartiles and min-max values) (**d** and **e**). n.s. (no significant); *p < 0.05, ****p < 0.0001. Source data are provided as a Source Data file.

References

1. Lavin Y*, et al.* Tissue-resident macrophage enhancer landscapes are shaped by the local microenvironment. *Cell* **159**, 1312-1326 (2014).

2. Buechler MB*, et al.* A Stromal Niche Defined by Expression of the Transcription Factor WT1 Mediates Programming and Homeostasis of Cavity-Resident Macrophages. *Immunity* **51**, 119-130 e115 (2019).

3. Okabe Y, Medzhitov R. Tissue-specific signals control reversible program of localization and functional polarization of macrophages. *Cell* **157**, 832-844 (2014).

4. Rosas M*, et al.* The transcription factor Gata6 links tissue macrophage phenotype and proliferative renewal. *Science* **344**, 645-648 (2014).

5. Gautier EL*, et al.* Gata6 regulates aspartoacylase expression in resident peritoneal macrophages and controls their survival. *J Exp Med* **211**, 1525-1531 (2014).

6. Kim KW*, et al.* MHC II+ resident peritoneal and pleural macrophages rely on IRF4 for development from circulating monocytes. *J Exp Med* **213**, 1951-1959 (2016).

7. Bain CC*, et al.* Long-lived self-renewing bone marrow-derived macrophages displace embryo-derived cells to inhabit adult serous cavities. *Nat Commun* **7**, ncomms11852 (2016).

8. Mass E*, et al.* Specification of tissue-resident macrophages during organogenesis. *Science* **353**, (2016).

9. Menendez-Gutierrez MP*, et al.* Retinoid X receptors orchestrate osteoclast differentiation and postnatal bone remodeling. *J Clin Invest* **125**, 809-823 (2015).

Supplementary Table 1: RNA-seq expression values for transcripts downregulated ≥1.5 FC in *LysM* $^{Cre+}$ *Rxra* $^{fl/fl}$ over *Rxra* $^{fl/fl}$ peritoneal LPMs.

ID	AvrExp	Norm_RXRKO2	Raw_RXRKO2	Norm_RXRKO3	Raw_RXRKO3	Norm_RXRWT2	Raw_RXRWT2	Norm_RXRWT3	Raw_RXRWT3	ave_KO	ave_WT	foldChange	logFC	P.Value	adj.P.Val
Galnt12	8.269946762	0.752814919	0	0.496586044	0	20.43982034	13	11.39056575	10	0.624700482	15.91519304	-23.95155619	-4.582047489	0.00008340	0.00548956
Asz1	28.20526942	2.258444757	1	2.482930222	2	59.80540025	39	48.27430246	44	2.37068749	54.03985135	-22.30820525	-4.479562545	0.00000084	0.00009885
Ncaph	15.08860035	5.269704432	3	0.496586044	0	28.01012417	18	26.57798675	24	2.883145238	27.29405546	-19.22178224	-4.264670204	0.00003246	0.00250431
Cyp26a1	10.38088915	2.258444757	1	0.496586044	0	21.9538811	14	16.81464468	15	1.377515401	19.38426289	-18.52312658	-4.211255732	0.00018289	0.01097588
Cdca2	9.337703292	0.752814919	0	1.489758133	1	12.86951651	8	22.23872361	20	1.121286526	17.55412006	-16.30159018	-4.026940798	0.00037520	0.01940870
Suv39h1	7.603168368	0.752814919	0	1.489758133	1	11.35545574	7	16.81464468	15	1.121286526	14.08505021	-13.18630325	-3.72096826	0.00114802	0.04842547
Traf3ip3	15.94384212	3.764074595	2	1.489758133	1	18.92575957	12	39.59577618	36	2.626916364	29.26076787	-12.83705293	-3.682242128	0.00007523	0.00509775
Ccnd1	109.7935593	20.32600281	13	15.39416738	15	171.8458969	113	231.6081702	213	17.86008509	201.7270336	-11.68527855	-3.54662022	0.00000000	0.00000000
Trib2	16.71373984	2.258444757	1	3.476102311	3	20.43982034	13	40.68059196	37	2.867273534	30.56020615	-10.44735715	-3.385066128	0.00013176	0.00828664
Gm1631	64.28959207	12.79785362	8	13.4078232	13	106.741284	70	124.2114075	114	13.10283841	115.4763457	-8.812270062	-3.139513709	0.00000000	0.00000001
Ccnb2	38.97090766	8.280964108	5	6.455618578	6	31.0382457	20	110.1088022	101	7.368291343	70.57352397	-8.730342094	-3.126038186	0.00000019	0.00002593
Slpi	139.1256022	30.86541168	20	27.31223245	27	223.3239629	147	275.0008017	253	29.08882206	249.1623823	-8.687514403	-3.118943465	0.00000000	0.00000000
Ptgis	298.8558667	60.97800843	40	66.04594392	66	397.440951	262	670.9585634	618	63.51197617	534.1997572	-8.266276546	-3.04723763	0.00000000	0.00000000
Gbp2b	316.7641845	86.57371568	57	54.12787885	54	318.7097912	210	807.6453524	744	70.35079726	563.1775718	-7.90569881	-2.982892993	0.00000000	0.00000000
Stmn1	83.64184552	29.35978184	19	11.42147902	11	64.34758254	42	229.4385387	211	20.39063043	146.8930606	-7.590240141	-2.92414553	0.00000000	0.00000000
Iigp1	38.39251734	3.764074595	2	15.39416738	15	34.06636723	22	100.3454602	92	9.579120987	67.2059137	-7.097628596	-2.827337083	0.00000172	0.00018664
Cenpe	62.66333633	17.31474314	11	12.41465111	12	52.23509642	34	168.6888547	155	14.86469712	110.4619755	-6.949145953	-2.796835682	0.00000000	0.00000019
Garnl3	56.11023176	15.8091133	10	13.4078232	13	84.0303725	55	111.193618	102	14.60846825	97.61199526	-6.8207802	-2.769936772	0.00000000	0.00000026
Mki67	393.3536325	127.2257213	84	86.90255779	87	417.123741	275	942.1625098	868	107.0641395	679.6431254	-6.287375261	-2.652457873	0.00000000	0.00000000
Ap1s1	17.95890179	12.79785362	8	2.482930222	2	23.46794187	15	33.08688146	30	7.640391922	28.27741167	-5.941719224	-2.570880432	0.00021485	0.01252539
Ubash3a	24.41941828	5.269704432	3	9.435134845	9	40.12261029	26	42.85022353	39	7.352419639	41.48641691	-5.579774265	-2.480206758	0.00006467	0.00449846
Cd24a	64.54467373	18.82037297	12	21.35319991	21	84.0303725	55	133.9747495	123	20.08678644	109.002561	-5.347122716	-2.418762787	0.00000000	0.00000074
Tha1	24.09208152	14.30348346	9	4.4692744	4	24.98200263	16	52.6135656	48	9.38637893	38.79778412	-5.284932926	-2.401885162	0.00006098	0.00429939
Tk1	18.30377	8.280964108	5	4.4692744	4	21.9538811	14	38.51096039	35	6.375119254	30.23242075	-5.262183412	-2.395661534	0.00037123	0.01931651
St14	18.09827091	5.269704432	3	6.455618578	6	26.4960634	17	34.17169725	31	5.862661505	30.33388032	-5.137383441	-2.361033757	0.00053467	0.02608888
Marco	921.3228354	207.0241027	137	398.7585937	401	1705.589453	1126	1373.919193	1266	302.8913482	1539.754323	-5.113862476	-2.354413364	0.00000000	0.00000000
Lrrk2	56.6320574	21.83163265	14	16.38733947	16	74.9460079	49	113.3632496	104	19.10948606	94.15462875	-5.085806564	-2.346476591	0.00000003	0.00000450
Top2a	92.68419609	23.33726249	15	31.2849208	31	70.40382561	46	245.7107754	226	27.31109164	158.0573005	-5.018680879	-2.327308213	0.00000000	0.00000009
Ccdc34	16.00813172	5.269704432	3	5.462446489	5	23.46794187	15	29.8324341	27	5.366075461	26.65018799	-4.99043569	-2.319165776	0.00109707	0.04685259
Nt5e	40.39094219	17.31474314	11	11.42147902	11	55.26321795	36	77.56432867	71	14.36811108	66.41377331	-4.909589517	-2.295602408	0.00000129	0.00014417
Gata6	38.60175294	5.269704432	3	24.33271618	24	53.74915718	35	71.05543396	65	14.80121031	62.40229557	-4.821941274	-2.26961408	0.00000888	0.00079175
Ltbp1	1143.855171	469.0036945	311	338.1750963	340	1816.115888	1199	1952.126006	1799	403.5893954	1884.120947	-4.818520068	-2.268590113	0.00000000	0.00000000
Prtg	36.52414307	27.854152	18	6.455618578	6	53.74915718	35	58.03764453	53	17.15488529	55.89340086	-4.758384281	-2.250471787	0.00000387	0.00039234
Rtel1	18.57775061	14.30348346	9	3.476102311	3	31.0382457	20	25.49317096	23	8.889792885	28.26570833	-4.515550152	-2.174901769	0.00067903	0.03204725
Itga6	921.3993852	369.6321252	245	321.2911708	323	1425.488211	941	1569.186034	1446	345.461648	1497.337122	-4.386970014	-2.133224845	0.00000000	0.00000000
Aspm	37.45634439	14.30348346	9	14.40099529	14	52.23509642	34	68.88580239	63	14.35223937	60.5604494	-4.235504925	-2.082533967	0.00001132	0.00098542
Pard3b	71.84659061	21.83163265	14	35.25760916	35	108.2553447	71	122.0417759	112	28.5446209	115.1485603	-4.011665068	-2.004201161	0.00000008	0.00001139
Vnn3	110.3925526	42.91045038	28	43.2029587	43	117.3397093	77	238.1170649	219	43.05671812	177.7283871	-3.994808292	-1.998126271	0.00000000	0.00000018
Thbs1	215.6490895	137.7651302	91	55.12105094	55	368.6737964	243	301.0363805	277	96.44309055	334.8550885	-3.99431457	-1.997947956	0.00000000	0.00000000
Itgb7	722.1124567	210.0353624	139	345.1273009	347	680.5703142	449	1652.716849	1523	277.5813316	1166.643582	-3.983363764	-1.993987234	0.00000000	0.00000000
Klra2	25.31437898	6.77533427	4	12.41465111	12	52.23509642	34	29.8324341	27	9.594992691	41.03376526	-3.943220117	-1.979374246	0.00048483	0.02399824
Naip1	331.8151153	122.7088318	81	146.4928831	147	514.02363	339	544.0351165	501	134.6008575	529.0293732	-3.916229644	-1.969465366	0.00000000	0.00000000
Knstrn	29.33123686	11.29222378	7	12.41465111	12	35.58042799	23	58.03764453	53	11.85343745	46.80903626	-3.908457082	-1.966599196	0.00013668	0.00853730
Cav1	56.85976818	27.854152	18	20.36002782	20	65.86164331	43	113.3632496	104	24.10708991	89.61244645	-3.815102516	-1.931721824	0.00000119	0.00013519
Fabp5	274.067218	109.1581632	72	118.6840646	119	445.8908955	294	422.5357485	389	113.9211139	434.213322	-3.788210918	-1.921516659	0.00000000	0.00000000
Khk	241.153273	103.1356439	68	96.83427868	97	259.6614213	171	504.9817482	465	99.98496128	382.3215848	-3.732433805	-1.900116674	0.00000000	0.00000000
Plekha6	47.64500014	8.280964108	5	36.25078125	36	59.80540025	39	86.24285496	79	22.26587268	73.0241276	-3.721954891	-1.89606057	0.00001761	0.00146975
Efcab5	87.42695227	33.87667135	22	39.23029751	39	149.1349854	98	127.4658548	117	36.55348443	138.3004201	-3.717423137	-1.894302913	0.00000004	0.00000572
Cenpf	80.01548357	21.83163265	14	42.20981378	42	68.88976484	45	187.130723	172	32.02072322	128.0102439	-3.716066028	-1.893776136	0.00000021	0.00002867
Wnt2	51.6374444	29.35978184	19	18.37368365	18	70.40382561	46	88.41248653	81	23.86673274	79.40815607	-3.566028163	-1.834318097	0.00000544	0.00052610
Derl1	48.62970096	17.31474314	11	25.32588827	25	68.88976484	45	82.9884076	76	21.3203157	75.93908622	-3.530486842	-1.81986714	0.00001245	0.00106354
St3gal5	62.23403008	29.35978184	19	26.31906036	26	88.57255479	58	104.6847233	96	27.8394211	96.62863905	-3.520073158	-1.815605413	0.00000160	0.00017702
Tlr13	260.5651058	160.3495777	106	85.9093857	86	362.6175534	239	433.3839064	399	123.1294817	398.0007299	-3.517829582	-1.814685594	0.00000000	0.00000000
Adgb	263.6405747	130.236981	86	107.7591717	108	377.758161	249	438.8079853	404	118.9980763	408.2830732	-3.492517436	-1.804267318	0.00000000	0.00000000
Hspa12a	69.63523559	50.43859957	33	20.36002782	20	105.2272232	69	102.5150917	94	35.3993137	103.8711575	-3.465429909	-1.793034339	0.00000078	0.00009318
2410016O06Rik	34.3465992	20.32600281	13	12.41465111	12	47.69291412	31	56.95282875	52	16.37032696	52.32287143	-3.454837551	-1.788617876	0.00012311	0.00776933
Fabp4	325.3344343	146.7989092	97	145.499711	146	509.4814477	336	499.5576693	460	146.1493101	504.5195585	-3.450877599	-1.786963303	0.00000000	0.00000000
Efnb2	375.5615391	167.8777269	111	176.2880458	177	583.6704252	385	574.4099585	529	172.0828864	579.0401918	-3.355344664	-1.746460969	0.00000000	0.00000000
Rad51b	26.53640973	18.82037297	12	8.441962756	8	29.52418493	19	49.35911825	45	13.63116786	39.44165159	-3.350901909	-1.744549455	0.00069321	0.03247883

Sla	100.0977355	50.43859957	33	43.20298587	43	152.163107	100	154.5862495	142	46.82079272	153.3746782	-3.321650262	-1.731900179	0.00000008	0.00001139
Mcemp1	226.9816757	94.10186486	62	116.6977205	117	342.9347634	226	354.192354	326	105.3997927	348.5635587	-3.290894172	-1.718479633	0.00000000	0.00000001
Kif20b	28.88981701	15.8091133	10	12.41465111	12	32.55230646	21	54.78319717	50	14.1118822	43.66775182	-3.169908288	-1.664441101	0.00074011	0.03397846
Zcwpw1	47.96079129	23.33726249	15	23.33954409	23	64.34758254	42	80.81877603	74	23.33840329	72.58317929	-3.123717642	-1.643264052	0.00005224	0.00376218
Mlxipl	117.5542912	32.37104151	21	81.93669734	82	111.2834663	73	244.6259597	225	57.15386943	177.954713	-3.116728668	-1.640032564	0.00000018	0.00002410
Alox15	321.2821701	181.4283955	120	137.5543343	138	488.2845969	322	477.8613536	440	159.4913649	483.0729753	-3.098849048	-1.63173248	0.00000000	0.00000000
H2-Ab1	904.9963106	160.3495777	106	610.3042487	614	365.6456749	241	2483.685741	2289	385.3269132	1424.665708	-3.080096378	-1.622975494	0.00000000	0.00000000
Tm2d1	50.52877397	29.35978184	19	21.35319991	21	82.51631173	54	68.88580239	63	25.35649088	75.70105706	-3.062455338	-1.614688804	0.00004810	0.00350544
Dennd4a	142.326519	112.1694229	74	46.18250214	46	202.1271122	133	208.8270387	192	79.17596253	205.4770755	-3.011540126	-1.590501482	0.00000004	0.00000595
Dock5	114.7899722	57.96674876	38	57.10739512	57	146.1068639	96	197.9788809	182	57.53707194	172.0428724	-2.995840666	-1.582960896	0.00000024	0.00003174
Arhgap24	39.53289346	23.33726249	15	17.38051156	17	58.29133948	38	59.12246032	54	20.35888702	58.7068999	-2.988971512	-1.579649147	0.00024644	0.01414266
Gm4951	62.94877839	24.84289232	16	38.23712543	38	84.0303725	55	104.6847233	96	31.54000888	94.35754791	-2.974405853	-1.572601514	0.00002110	0.00171417
Rab27a	257.8713467	157.3383181	104	113.7182042	114	359.5894319	237	400.8394328	369	135.5282611	380.2144323	-2.899703631	-1.535905454	0.00000000	0.00000010
Serpinb2	138.9753468	104.6412737	69	50.15519049	50	159.7334108	105	241.3715123	222	77.39823211	200.5524615	-2.877508931	-1.524820406	0.00000014	0.00002015
Gbp3	371.0534084	176.9115059	117	203.1036922	204	397.440951	262	706.7574843	651	190.0075991	552.0992177	-2.840355776	-1.506070634	0.00000000	0.00000001
Dusp1	36.86127216	23.33726249	15	12.41465111	12	84.0303725	55	27.66280253	25	17.8759568	55.84658752	-2.81637576	-1.493839831	0.00117127	0.04879294
Adap1	34.34265111	12.79785362	8	22.346372	22	58.29133948	38	43.93503932	40	17.57211281	51.1131894	-2.811565437	-1.491373625	0.00115236	0.04842547
Mcm5	56.49711794	39.8991907	26	23.33954409	23	61.31946101	40	101.430276	93	31.6193674	81.37486848	-2.75616141	-1.46266038	0.00010407	0.00670592
Aifm2	60.32723474	18.82037297	12	46.18250214	46	97.65691939	64	78.64914446	72	32.50143756	88.15303192	-2.747057834	-1.457887284	0.00010600	0.00678240
Rps16	44.33159622	20.32600281	13	26.31906036	26	47.69291412	31	82.9884076	76	23.32253158	65.34066086	-2.737842261	-1.453039329	0.00042897	0.02164155
Ranbp1	63.9049369	32.37104151	21	32.27809289	32	126.4240739	83	64.54653925	59	32.3245672	95.48530659	-2.711144041	-1.438901765	0.00008801	0.00577250
Pycard	2372.474253	871.0068612	578	1756.424839	1768	2972.858313	1963	3889.606999	3585	1313.71585	3431.232656	-2.684541799	-1.424675868	0.00000000	0.00000000
Mcm3	41.47736388	30.86541168	20	17.38051156	17	47.69291412	31	69.97061817	64	24.12296162	58.83176615	-2.664346686	-1.413781819	0.00068059	0.03204725
Plxdc2	1314.202442	762.6015129	506	683.7989833	688	1861.537711	1229	1948.871559	1796	723.2002481	1905.204635	-2.65694487	-1.409768293	0.00000000	0.00000000
Cav2	49.72170627	32.37104151	21	23.33954409	23	76.46006867	50	66.71617082	61	27.8552928	71.58811974	-2.648474745	-1.405161752	0.00031718	0.01718221
Ppp1r9a	49.96067916	32.37104151	21	21.35319991	21	37.09448876	24	109.0239865	100	26.86212071	73.05923761	-2.636592644	-1.398674691	0.00045946	0.02290755
Selp	782.2169716	399.7447219	265	462.3216074	465	1062.113627	701	1204.68793	1110	431.0331647	1133.400779	-2.625824599	-1.392770549	0.00000000	0.00000000
Mthfd1	44.67130524	18.82037297	12	30.29174871	30	55.26321795	36	74.30988132	68	24.55606084	64.78654963	-2.620923281	-1.390075124	0.00066753	0.03177025
Cfb	453.5247013	201.0015834	133	269.6462222	271	345.962885	228	997.4881149	919	235.3239028	671.7254999	-2.593147081	-1.374704037	0.00000000	0.00000007
Tacc3	51.28490839	29.35978184	19	27.31223245	27	49.20697489	32	99.26064439	91	28.33600714	74.23380964	-2.567280043	-1.360240676	0.00046016	0.02290755
Mcm2	56.98695568	30.86541168	20	33.27126498	33	68.88976484	45	94.92138124	87	32.06833833	81.90557304	-2.546833177	-1.348704463	0.00028509	0.01586491
Smc4	270.5233187	112.1694229	74	195.1583155	196	283.8863936	187	490.879143	452	153.6638692	387.3827683	-2.504918799	-1.324763837	0.00000005	0.00000810
Pilra	188.7062836	86.57371568	57	129.6089576	130	235.4364491	155	303.2060121	279	108.0913366	269.3212306	-2.491857411	-1.317221517	0.00000063	0.00007664
Abr	88.31977561	56.46111892	37	46.18250214	46	126.4240739	83	124.2114075	114	51.32181053	125.3177407	-2.484246478	-1.31280832	0.00004725	0.00345698
Stk10	179.918857	74.52867697	49	135.5679901	136	232.4083476	153	277.1704332	255	105.0483336	254.7893804	-2.459952564	-1.298630496	0.00000125	0.00014171
Enpp4	98.9554355	56.46111892	37	58.10056721	58	115.8256486	76	165.4344073	152	57.28084306	140.6300279	-2.449335473	-1.292390386	0.00003576	0.00273616
Mpeg1	78.48862837	63.98926811	42	33.27126498	33	87.05849403	57	129.6354864	119	48.63026654	108.3469902	-2.447771795	-1.291469062	0.00011351	0.00718819
Rpl12	149.3778313	60.97800843	40	116.6977205	117	206.6692945	136	213.1663019	196	88.83786444	209.9177982	-2.400912586	-1.263582878	0.00000676	0.00062984
Dock10	95.40068359	80.55119632	53	40.2234696	40	127.9381347	84	132.8899337	122	60.38733296	130.4140342	-2.396555295	-1.260962227	0.00006220	0.00434295
Klhl6	408.2330681	261.2267769	173	222.967134	224	447.4049563	295	701.3334054	646	242.0969554	574.3691808	-2.381027464	-1.251584261	0.00000001	0.00000165
Tap1	83.48850412	33.87667135	22	64.05959974	64	82.51631173	54	153.5014337	141	48.96813555	118.0088727	-2.37972966	-1.250797691	0.00016499	0.01003250
Icam2	849.6049176	465.9924348	309	538.7958583	542	1092.394842	721	1301.236535	1199	502.3941465	1196.815689	-2.379214989	-1.250485641	0.00000000	0.00000000
Gm16548	298.2198337	154.3270584	102	197.1446597	198	342.9347634	226	498.4728535	459	175.735859	420.7038085	-2.375798621	-1.248412555	0.00000011	0.00001577
Nrip1	85.42678352	47.42733989	31	49.1620184	49	156.7052893	103	88.41248653	81	48.29467915	122.5588879	-2.371892952	-1.2460389	0.00014656	0.00909274
Fam212a	73.40435198	35.38230119	23	51.14836258	51	106.741284	70	100.3454602	92	43.26533189	103.5433721	-2.365756266	-1.242301446	0.00026907	0.01515728
Mtus1	204.7915835	94.10186486	62	139.5406785	140	370.1878572	244	215.3359334	198	116.8212717	292.7618953	-2.360910396	-1.239343288	0.00000184	0.00019738
Flnb	543.725743	331.9913792	220	318.3116545	320	701.7671649	463	822.8327734	758	325.1515169	762.2999692	-2.357242836	-1.237100389	0.00000000	0.00000022
Tgfb2	941.7704046	746.0395846	495	424.581068	427	1383.094509	913	1213.366456	1118	585.3103263	1298.230483	-2.353994356	-1.235110861	0.00000000	0.00000000
Rpl13a	68.17483935	30.86541168	20	49.1620184	49	67.37570407	44	125.2962232	115	40.01371504	96.33596366	-2.347827268	-1.231326272	0.00046006	0.02290755
Irf1	164.8675085	88.07934551	58	98.82062285	99	132.480317	87	340.0897488	313	93.44998418	236.2850329	-2.347249457	-1.230971174	0.00000809	0.00072793
P4ha2	55.08097022	50.43859957	33	22.346372	22	68.88976484	45	78.64914446	72	36.39248578	73.76945465	-2.343306882	-1.228545903	0.00094542	0.04136297
Itgam	2916.36155	1405.505454	933	2102.048726	2116	3623.904443	2393	4533.987576	4179	1753.77709	4078.946009	-2.332692063	-1.221995871	0.00000000	0.00000000
Ezh2	83.84408484	38.39356086	25	62.07325556	62	90.08661556	59	144.8229074	133	50.23340821	117.4547615	-2.314904709	-1.210952807	0.00022745	0.01317640
Pilrb2	80.80444058	48.93296973	32	49.1620184	49	97.65691939	64	127.4658548	117	49.04749407	112.5613871	-2.30082084	-1.202148648	0.00025813	0.01463661
Eif4e3	77.14321052	39.8991907	26	53.1347067	53	109.7694055	72	105.7695391	97	46.51694873	107.7694723	-2.292133779	-1.196691249	0.00034328	0.01827264
Emilin2	574.860773	315.429451	209	382.8678403	385	715.3937118	472	885.752089	816	349.1486457	800.5729004	-2.288349333	-1.194307306	0.00000000	0.00000047
Net1	62.69805641	51.94422941	34	30.29174871	30	77.97412943	51	90.5821181	83	41.11798906	84.27812377	-2.215323817	-1.147517595	0.00117173	0.04879294
Kctd9	93.94430542	45.92171005	30	70.01863227	70	97.65691939	64	162.17996	149	57.97017116	129.9184397	-2.209252027	-1.143558008	0.00029938	0.01641132
Dnajc9	74.37905698	42.91045038	28	47.17567423	47	65.86164331	43	141.56846	130	45.0430623	103.7150517	-2.207138277	-1.142177017	0.00077146	0.03498066
Car6	72.22612345	21.83163265	14	72.00497645	72	64.34758254	42	130.7203022	120	46.91830455	97.53394236	-2.197498168	-1.135861963	0.00118460	0.04921713
Impdh2	200.8398275	98.61875438	65	154.4382598	155	253.6051783	167	296.6971174	273	126.5285071	275.1511478	-2.183430684	-1.126596731	0.00001217	0.00104956
Pelp1	212.1704619	112.1694229	74	154.4382598	155	292.9707582	193	289.1034069	266	133.3038414	291.0370825	-2.172909062	-1.119627798	0.00001009	0.00089123
Cgnl1	236.8977491	109.1581632	72	194.1651434	195	317.1957304	209	327.0719594	301	151.6616533	322.1338449	-2.149713578	-1.104144452	0.00000806	0.00072793

Stxbp2	322.7921322	185.945285	123	222.967134	224	464.0596247	306	418.1964854	385	204.4562095	441.128055	-2.135336448	-1.094463402	0.00000169	0.00018477
F10	393.0823011	169.3833568	112	342.1477847	344	470.1158678	310	590.6821953	544	255.7655707	530.3990315	-2.133477157	-1.093206664	0.00000063	0.00007664
Ldlr	253.907064	143.7876495	95	177.2812179	178	261.1754821	172	433.3839064	399	160.5344337	347.2796942	-2.126639074	-1.088575204	0.00000749	0.00069096
Ndufa1	178.999296	94.10186486	62	134.5748181	135	203.641173	134	283.6793279	261	114.3383415	243.6602505	-2.122421167	-1.085710969	0.00004069	0.00304974
Rpl23	2853.073068	1515.416432	1006	2158.659535	2173	3295.353257	2176	4442.86305	4095	1837.037984	3869.108153	-2.104458133	-1.073448808	0.00000000	0.00000000
Arg1	210.5884345	56.46111892	37	247.7964362	249	271.7739074	179	266.3222754	245	152.1287776	269.0480914	-2.098520364	-1.069372463	0.00003824	0.00288967
Ptgs1	447.0407227	207.0241027	137	377.9019799	380	574.5860606	379	628.6507478	579	292.4630413	601.6184042	-2.093585974	-1.065976164	0.00000050	0.00006339
Tapbpl	194.7432836	125.7200915	83	126.6294413	127	193.0427476	127	333.5808541	307	126.1747664	263.3118009	-2.054361935	-1.038690376	0.00005930	0.00422094
Bank1	114.9403712	103.1356439	68	55.12105094	55	164.2755931	108	137.2291969	126	79.12834741	150.752395	-2.049895479	-1.035550351	0.00046030	0.02290755
Rps8	977.6497335	550.3077057	365	732.4644156	737	1202.921278	794	1424.905534	1313	641.3860607	1313.913406	-2.047196459	-1.033649557	0.00000000	0.00000069
Lancl1	237.9932777	176.9115059	117	140.5338506	141	309.6254266	204	324.9023278	299	158.7226783	317.2638772	-2.039304389	-1.02807713	0.00002691	0.00213020
Lyz2	9617.431148	6386.128957	4241	6229.671928	6272	14263.20944	9420	11590.71426	10684	6307.900443	12926.96185	-2.021265367	-1.015258742	0.00000000	0.00000000
Tcf7l2	143.0620412	79.04556649	52	108.7523437	109	143.0787424	94	241.3715123	222	93.89895512	192.2251273	-2.010937376	-1.007868155	0.00031493	0.01711084
Cotl1	500.8888301	255.2042575	169	417.6288634	420	603.3532151	398	727.3689843	670	336.4165605	665.3610997	-1.995522572	-0.996766598	0.00000126	0.00014171
Rpl31	185.4796181	103.1356439	68	145.499711	146	239.9786314	158	253.3044859	233	124.3176775	246.6415587	-1.980123058	-0.985590092	0.00015504	0.00953616
Pygl	483.8916824	294.3506333	195	358.0385381	360	542.7907845	358	740.3867737	682	326.1945857	641.5887791	-1.957999177	-0.969380158	0.00000275	0.00028532
H2-T23	743.7484391	473.520584	314	522.9051049	526	745.6749271	492	1232.89314	1136	498.2128444	989.2840337	-1.956462578	-0.968247516	0.00000023	0.00003037
Laptm5	446.7751908	264.2380365	175	339.1682684	341	509.4814477	336	674.2130108	621	301.7031525	591.8472292	-1.955424605	-0.967481911	0.00000440	0.00043864
Rpl21	660.1710005	372.6433849	247	521.9119328	525	698.7390434	461	1047.389641	965	447.2776588	873.0643422	-1.939124284	-0.955405273	0.00000068	0.00008252
Adipor2	1991.868471	1236.874912	821	1470.391278	1480	2617.054034	1728	2643.153662	2436	1353.633095	2630.103848	-1.934054515	-0.95162846	0.00000000	0.00000002
Rpl5	6188.814456	3322.172237	2206	5122.285049	5157	6824.628901	4507	9486.171637	8744	4222.228643	8155.400269	-1.93399313	-0.95158267	0.00000000	0.00000000
F5	1312.673881	783.6803306	520	1010.552601	1017	1676.822298	1107	1779.640296	1640	897.1164656	1728.231297	-1.921431205	-0.942181323	0.00000001	0.00000123
Rgs10	417.6068197	246.1704785	163	325.2638591	327	467.0877462	308	631.9051951	582	285.7171688	549.4964707	-1.91653897	-0.938503333	0.00001152	0.00099807
Fam46a	2089.800997	1259.459359	836	1603.476338	1614	2615.539973	1727	2880.728319	2655	1431.467849	2748.134146	-1.915833337	-0.937972063	0.00000000	0.00000002
Lbr	268.9470649	175.4058761	116	193.1719713	194	309.6254266	204	397.5849854	366	184.2889237	353.605206	-1.915592418	-0.93779063	0.00007686	0.00517265
Serpinb1a	201.7455649	160.3495777	106	121.6635809	122	236.9505098	156	288.0185911	265	141.0065793	262.4845505	-1.912302127	-0.935310475	0.00023259	0.01343146
Clec4e	349.4416182	217.5635116	144	266.6667059	268	451.9471386	298	461.5891168	425	242.1151087	456.7681277	-1.878873163	-0.909867678	0.00004348	0.00323267
Emr1	485.4614914	404.2616115	268	278.584771	280	713.879651	471	545.1199323	502	341.4231912	629.4997917	-1.873004683	-0.905354507	0.00001126	0.00098542
Psmb9	158.8981374	112.1694229	74	105.7728275	106	141.5646816	93	276.0856174	254	108.9711252	208.8251495	-1.870327434	-0.903290861	0.00086552	0.03839219
Rpl18a	1203.862993	694.8481702	461	981.75061	988	1317.989897	870	1820.863296	1678	838.2993901	1569.426596	-1.868177959	-0.90163189	0.00000006	0.00000863
Igsf6	304.3539231	235.6310696	156	194.1651434	195	336.8785204	222	450.7409589	415	214.8981065	393.8097396	-1.860520982	-0.895706661	0.00009759	0.00631056
Fblim1	155.3247135	128.7313511	85	91.86841823	92	150.6490462	99	250.0500386	230	110.2998847	200.3495424	-1.855356036	-0.891696062	0.00103421	0.04427079
Irak1	195.0376517	118.1919423	78	155.4314319	156	236.9505098	156	269.5767227	248	136.8116871	253.2636163	-1.84737148	-0.885474001	0.00054429	0.02644294
Trf	4168.561419	3114.39532	2068	2718.808594	2737	6231.117081	4115	4609.924681	4249	2916.601957	5420.520881	-1.830552615	-0.872279241	0.00000000	0.00000000
Prg4	22451.99059	15129.32143	10048	16566.60703	16680	28428.76197	18776	29683.27193	27362	15847.96423	29056.01695	-1.828273672	-0.870482041	0.00000000	0.00000000
Cd5l	1854.472909	1009.524806	670	1623.339779	1634	2486.844808	1642	2298.182242	2118	1316.432293	2392.513525	-1.81720966	-0.86172488	0.00000001	0.00000139
C4b	1166.483703	592.4653412	393	1091.992712	1099	1202.921278	794	1778.555481	1639	842.2290265	1490.738379	-1.792381899	-0.841878062	0.00000052	0.00006458
Rps25	1536.5376	833.3661152	553	1393.917027	1403	1658.653569	1095	2260.213689	2083	1113.641571	1959.433629	-1.773273687	-0.826415219	0.00000013	0.00001767
Psmb8	331.030062	164.8664672	109	300.4345569	302	255.119239	168	603.6999847	556	232.6505121	429.4096119	-1.770702614	-0.824321934	0.00030858	0.01681517
Rpl22	242.4508902	133.2482406	88	218.9944456	220	253.6051783	167	363.9556961	335	176.1213431	308.7804372	-1.759080682	-0.814821655	0.00080685	0.03631594
Saa3	285.9648216	139.27076	92	282.5574593	284	358.0753711	236	363.9556961	335	210.9141097	361.0155336	-1.757865744	-0.81382489	0.00050705	0.02496359
Rpl4	2295.874038	1610.271112	1069	1723.65016	1735	2656.419613	1754	3193.155265	2943	1666.960636	2924.787439	-1.755739803	-0.812079056	0.00000001	0.00000143
Fam69a	677.9498093	393.7222026	261	592.4271511	596	725.9921372	479	999.6577465	921	493.0746768	862.8249418	-1.751106153	-0.808266543	0.00002126	0.00172015
Eef1b2	1204.905374	756.5789935	502	987.7096425	994	1231.688433	813	1843.644428	1699	872.144318	1537.66643	-1.748732692	-0.806309779	0.00000111	0.00012780
Htatsf1	231.5302855	170.8889866	113	168.3426691	169	279.3442113	184	307.5452752	283	169.6158278	293.4447432	-1.73598836	-0.795757275	0.00112725	0.04780806
Itgb1	339.7015974	256.7098873	170	239.8510595	241	339.9066419	224	522.3388008	481	248.2804734	431.1227213	-1.732768669	-0.793079062	0.00038805	0.01979476
Rpl3	4958.715875	2890.056474	1919	4383.365015	4413	5637.605261	3723	6923.836752	6382	3636.710744	6280.721006	-1.732328341	-0.7927124	0.00000000	0.00000000
Engase	350.8359349	231.1141801	153	279.577943	281	470.1158678	310	422.5357485	389	255.3460616	446.3258081	-1.729744324	-0.790558807	0.00035843	0.01891431
Npm1	1293.259135	843.9055241	560	1059.218033	1066	1313.447714	867	1956.465269	1803	951.5617785	1634.956492	-1.703924046	-0.768861027	0.00000222	0.00023581
Ppm1h	314.4081263	226.5972906	150	238.8578874	240	406.5253156	268	385.6520118	355	232.727589	396.0886637	-1.694008003	-0.76044069	0.00080462	0.03630479
Pabpc1	9378.292128	5990.14831	3978	7971.695772	8026	10113.16888	6679	13438.15554	12387	6980.922041	11775.66221	-1.682427733	-0.750544537	0.00000000	0.00000000
Rpl11	533.627306	301.8787825	200	506.0211793	509	600.3250936	396	726.2841685	669	403.9499809	663.304631	-1.659737802	-0.730955349	0.00027310	0.01533691
Sod1	443.6823757	331.9913792	220	333.2092359	335	427.7221663	282	681.8067213	628	332.6003075	554.7644438	-1.653431569	-0.725463338	0.00053559	0.02608888
Rps3	3738.670016	2369.10855	1573	3291.868889	3314	4393.047312	2901	4900.655312	4517	2830.488719	4646.851312	-1.640677054	-0.714291291	0.00000001	0.00000153
Cybb	699.5445361	532.2401477	353	531.8436537	535	801.6951754	529	932.3991678	859	532.0419007	867.0471176	-1.634896224	-0.709199063	0.00015426	0.00953616
Rpsa	2869.514415	1940.004046	1288	2425.822827	2442	3310.493864	2186	3801.736921	3504	2182.913437	3556.115392	-1.62608557	-0.701403179	0.00000013	0.00001767
Fn1	7640.561254	5898.30489	3917	5938.672506	5979	9949.650322	6571	8775.617298	8089	5918.488698	9362.63381	-1.569084548	-0.649923092	0.00000000	0.00000005
Sdf4	1319.492357	956.8277619	635	1094.972228	1102	1637.456718	1081	1588.712718	1464	1025.899995	1613.084718	-1.563234053	-0.6445338	0.00006019	0.00426742
Rpl7	716.9835005	539.7682969	358	583.4886023	587	747.1889879	493	997.4881149	919	561.6284496	872.3385514	-1.550195909	-0.632450551	0.00067966	0.03204725
Msr1	704.8514212	568.3752638	377	546.741235	550	757.7874132	500	946.501773	872	557.5582494	852.1445931	-1.535681887	-0.618879396	0.00091665	0.04027115
Notch2	698.8463556	509.6557001	338	593.4203232	597	772.9280209	510	919.3813783	847	551.5380116	846.1546996	-1.531966171	-0.61538444	0.00100621	0.04337582
Rps14	835.2712684	524.7119985	348	800.0001177	805	1009.1215	666	1007.251457	928	662.3560581	1008.186479	-1.523895542	-0.607764014	0.00071630	0.03321753

Supplementary Table 2: RNA-seq expression values for transcripts upregulated ≥1.5 FC in *LysM* $^{Cre+}$ *Rxra* $^{fl/fl}$ over *Rxra* $^{fl/fl}$ peritoneal LPMs.

ID	AvrExp	Norm_RXRKO2	Raw_RXRKO2	Norm_RXRKO3	Raw_RXRKO3	Norm_RXRWT2	aw_RXRW	orm_RXRW	Raw_RXRWT3	ave_KO	ave_WT	foldChange	logFC	P.Value	adj.P.Val
Cd209f	1035.261132	2649.1557	1759	1474.363966	1484	15.89763804	10	1.6272237	1	2061.759833	8.762430859	450.5583092	8.815570015	0.00000000	0.00000000
Mucl1	113.8429065	324.4632301	215	129.6089576	130	0.757030383	0	0.5424079	0	227.0360938	0.649719138	305.7473736	8.256196296	0.00000000	0.00000000
Fcrls	619.8682687	1220.312984	810	1231.036804	1239	26.4960634	17	1.6272237	1	1225.674894	14.06164354	233.07623	7.86465807	0.00000000	0.00000000
Cd209g	191.7788592	482.554363	320	279.577943	281	2.271091149	1	2.7120395	2	381.066153	2.491565306	142.8232992	7.158087539	0.00000000	0.00000000
Cd209d	29.29407345	39.8991907	26	75.97766481	76	0.757030383	0	0.5424079	0	57.93842775	0.649719138	89.49642011	6.48375807	0.00000001	0.00000127
Cbr2	771.6498263	1795.463582	1192	1212.166535	1220	71.91788637	47	7.0513026	6	1503.815058	39.48459449	74.83798373	6.225698785	0.00000000	0.00000000
Tslp	44.52520474	110.6637931	73	65.05277183	65	0.757030383	0	1.6272237	1	87.85828245	1.192127031	73.02425631	6.190303855	0.00000001	0.00000241
Clec4b1	16.35902873	30.86541168	20	33.27126498	33	0.757030383	0	0.5424079	0	32.06833833	0.649719138	50.29188196	5.652253636	0.00000059	0.00007252
Gcnt1	48.29067105	125.7200915	83	60.08691138	60	6.813273446	4	0.5424079	0	92.90350142	3.677840669	48.86761433	5.61080677	0.00000001	0.00000123
Rnase4	421.7650745	1157.07653	768	490.1304259	493	21.9538811	14	17.89946	16	823.6034781	19.92667078	36.90836616	5.20587597	0.00000000	0.00000000
Lyve1	511.3652913	1108.896376	736	845.6860338	851	79.4881902	52	11.390566	10	977.2912047	45.43937797	35.83989523	5.163494515	0.00000000	0.00000000
Retnla	1195.022189	1589.192294	1055	3012.787532	3033	146.1068639	96	32.002066	29	2300.989913	89.05446478	35.60827106	5.154140483	0.00000000	0.00000000
Folr2	271.2073831	512.6669598	340	518.9324165	522	46.17885335	30	7.0513026	6	515.7996881	26.61507798	32.51339423	5.022962269	0.00000000	0.00000000
Fxyd2	90.51535632	250.687368	166	98.82062285	99	9.841394977	6	2.7120395	2	174.7539954	6.276717221	32.13700322	5.006163498	0.00000000	0.00000000
Prune2	154.9073252	429.8573187	285	173.3085295	174	8.327334211	5	8.1361184	7	301.5829241	8.231726302	31.76851558	4.989525775	0.00000000	0.00000000
Stac2	16.50211824	29.35978184	19	34.26443707	34	0.757030383	0	1.6272237	1	31.81210945	1.192127031	28.46407113	4.831070116	0.00001350	0.00114814
Hpgd	9.88753845	24.84289232	16	13.4078232	13	0.757030383	0	0.5424079	0	19.12535776	0.649719138	27.04233444	4.757147793	0.00003655	0.00278506
Cd163	600.1227177	1560.585327	1036	756.3005458	761	46.17885335	30	37.426145	34	1158.442936	41.80249898	25.50009362	4.672430639	0.00000000	0.00000000
Rab3il1	18.0774878	18.82037297	12	49.1620184	49	3.785151914	2	0.5424079	0	33.99119569	2.163779904	24.83663227	4.634397659	0.00001362	0.00115276
1700088E04Rik	7.124571797	12.79785362	8	14.40099529	14	0.757030383	0	0.5424079	0	13.59942446	0.649719138	21.40763534	4.420053541	0.00015527	0.00953616
Rcn3	124.7906065	285.3168543	189	187.2129388	188	17.41169881	11	9.2209342	8	236.2648965	13.31631649	18.70076593	4.225025455	0.00000000	0.00000000
F13a1	1098.992325	2442.884412	1622	1621.353435	1632	282.3723328	186	49.359118	45	2032.118924	165.8657255	17.80841623	4.154487313	0.00000000	0.00000000
Kazald1	28.13138469	41.40482054	27	65.05277183	65	2.271091149	1	3.7968552	3	53.22879618	3.033973199	17.71505362	4.146903927	0.00000177	0.00019155
Mrc1	468.5631794	1011.030436	671	739.4166203	744	96.14285862	63	27.662803	25	875.2235282	61.90283058	17.62192049	4.139299257	0.00000000	0.00000000
Gja1	9.857902774	11.29222378	7	25.32588827	25	2.271091149	1	0.5424079	0	18.30905603	1.406749521	16.80469567	4.07079251	0.00033435	0.01795329
Tfrc	231.9165328	351.5645671	233	513.9665561	517	43.15073182	28	18.984276	17	432.7655616	31.06750404	15.93496359	3.994123818	0.00000000	0.00000000
Ggh	6.13933556	15.8091133	10	7.448790667	7	0.757030383	0	0.5424079	0	11.62895198	0.649719138	15.82122309	3.98378923	0.00076765	0.03489370
Cd2	6.267449997	17.31474314	11	6.455618578	6	0.757030383	0	0.5424079	0	11.88518086	0.649719138	15.08861407	3.915388391	0.00096419	0.04195823
Ccr2	283.1512192	375.6546445	249	654.9969927	659	76.46006867	50	25.493171	23	515.3258186	50.97661982	12.26451008	3.6164177	0.00000000	0.00000000
Clec4n	19.88236815	26.34852216	17	43.20298587	43	0.757030383	0	9.2209342	8	34.77575402	4.98898228	11.67189366	3.544966739	0.00009190	0.00598426
Clec4g	32.9496089	63.98926811	42	56.11422303	56	6.813273446	4	4.881671	4	60.05174557	5.847472241	10.58437784	3.403864564	0.00000041	0.00005247
Slamf9	169.0414158	342.5307881	227	271.6325663	273	15.89763804	10	46.104671	42	307.0816772	31.00115446	10.40708159	3.379493652	0.00000000	0.00000000
Plk2	19.43619743	45.92171005	30	25.32588827	25	3.785151914	2	2.7120395	2	35.62379916	3.248595689	10.37660632	3.37526278	0.00008127	0.00536826
Zfp108	18.56549226	53.44985924	35	10.42830693	10	9.841394977	6	0.5424079	0	31.93908309	5.191901435	10.33121933	3.368938632	0.00041067	0.02083314
Acvrl1	16.42193339	26.34852216	17	33.27126498	33	2.271091149	1	3.7968552	3	29.80989357	3.033973199	9.994243506	3.321097369	0.00017396	0.01047437
Emp1	71.95164376	127.2257213	84	129.6089576	130	17.41169881	11	13.560197	12	128.4173395	15.48594806	8.513179991	3.089698133	0.00000000	0.00000000
Gm15987	16.25642803	18.82037297	12	36.25078125	36	8.327334211	5	1.6272237	1	27.53557711	4.977278945	8.281105629	3.049823398	0.00023552	0.01355801
Ophn1	21.18057178	41.40482054	27	32.27809289	32	8.327334211	5	2.7120395	2	36.84145672	5.519686838	8.231764328	3.041201679	0.00004232	0.00315937
Ccl2	16.84595596	45.92171005	30	15.39416738	15	2.271091149	1	3.7968552	3	30.65793872	3.033973199	8.087327815	3.015663091	0.00069330	0.03247883
Lmo2	29.51722071	65.49489795	43	40.2234696	40	5.29921268	3	7.0513026	6	52.85918377	6.175257643	7.952695597	2.99144395	0.00000591	0.00055610
Tanc2	33.78882156	91.09060519	60	31.2849208	31	6.813273446	4	5.9664868	5	61.187763	6.389880133	7.903860502	2.982557485	0.00000419	0.00042004
Plekho1	35.62355822	60.97800843	40	65.05277183	65	8.327334211	5	8.1361184	7	63.01539013	8.231726302	7.695469605	2.944009368	0.00000041	0.00005282
Trem2	16.82425613	35.38230119	23	24.33271618	24	3.785151914	2	3.7968552	3	29.85750868	3.791003582	7.518429387	2.910431312	0.00036167	0.01892232
Clec10a	48.71090969	97.11312454	64	74.98449272	75	11.35545574	7	11.390566	10	86.04880863	11.37301075	7.392230066	2.886009658	0.00000001	0.00000234
Pea15a	153.6474287	294.3506333	195	246.8032641	248	37.09448876	24	36.341329	33	270.5769487	36.71790879	7.286427648	2.865211671	0.00000000	0.00000000
Fcgr2b	44.70076011	54.95548908	36	99.81379494	100	15.89763804	10	8.1361184	7	77.38464201	12.01687822	7.080051015	2.823759756	0.00000004	0.00000572
Tmem8	23.2976332	32.37104151	21	42.20981378	42	15.89763804	10	2.7120395	2	37.29042765	9.304838752	6.602861596	2.723091406	0.00003084	0.00240968
Lgals1	27.16061166	48.93296973	32	45.18933005	45	5.29921268	3	9.2209342	8	47.06114989	7.260073429	6.406827761	2.679610206	0.00002032	0.00167300
Maf	313.0992007	578.9146726	384	493.1099422	496	115.8256486	76	64.546539	59	536.0123074	90.18609391	6.297404976	2.654757448	0.00000000	0.00000000
C1qtnf1	46.39566253	80.55119632	53	72.99814854	73	24.98200263	16	7.0513026	6	76.77467243	16.01665262	6.289924899	2.653042791	0.00000004	0.00000696
Egr2	89.32035516	249.1817382	165	58.10056721	58	38.60854953	25	11.390566	10	153.6411527	24.99955764	5.754558617	2.524705276	0.00000000	0.00000012
Npl	23.88507695	56.46111892	37	24.33271618	24	2.271091149	1	12.475382	11	40.39691755	7.373236342	5.730162461	2.518576043	0.00038606	0.01976463
Ccl6	1245.936862	1991.195461	1322	2232.15427	2247	439.8346524	290	320.56306	295	2111.674865	380.1988585	5.718594028	2.51566049	0.00000000	0.00000000
Stxbp6	27.14088571	67.00052778	44	25.32588827	25	11.35545574	7	4.881671	4	46.16320803	8.118563389	5.523075897	2.465471953	0.00006725	0.00464250
Marcks	205.4689428	360.5983462	239	322.2843429	324	96.14285862	63	42.850224	39	341.4413445	69.49654108	5.49671957	2.458570878	0.00000000	0.00000000
Rgl1	86.05822322	134.7538705	89	154.4382598	155	21.9538811	14	33.086881	30	144.5960652	27.52038128	5.251270332	2.392666466	0.00000000	0.00000004

Rnf150	55.50642465	100.1243842	66	82.92986943	83	26.4960634	17	12.475382	11	91.52712682	19.48572247	5.220504012	2.384189098	0.00000004	0.00000588
Sepp1	1364.019241	2454.929451	1630	2113.966791	2128	485.2564754	320	401.92425	370	2284.448121	443.590362	5.156264741	2.366326339	0.00000000	0.00000000
Eid1	27.18159546	60.97800843	40	31.2849208	31	8.327334211	5	8.1361184	7	46.13146462	8.231726302	5.080602662	2.34499964	0.00007826	0.00520724
Fam189b	26.30770532	50.43859957	33	37.24395334	37	8.327334211	5	9.2209342	8	43.84127645	8.774134195	4.808469394	2.265577736	0.00009583	0.00621875
Zdhhc14	114.8135085	163.3608374	108	214.0285852	215	47.69291412	31	34.171697	31	188.6947113	40.93230568	4.784508633	2.258370768	0.00000000	0.00000000
Slc9a9	54.92472226	101.6300141	67	74.98449272	75	29.52418493	19	13.560197	12	88.30725339	21.54219113	4.518265925	2.175769184	0.00000025	0.00003289
Ccl9	816.5603969	1202.245426	798	1464.432245	1474	320.2238519	211	279.34006	257	1333.338835	299.7819584	4.505110819	2.171562594	0.00000000	0.00000000
Tbc1d14	39.21489083	62.48363827	41	64.05959974	64	18.92575957	12	11.390566	10	63.271619	15.15816266	4.473229632	2.161316821	0.00000536	0.00052096
Ank2	40.78203361	63.98926811	42	69.02546018	69	14.38357727	9	15.729829	14	66.50736415	15.05670308	4.410639239	2.140987762	0.00000515	0.00050541
Anxa5	151.5133741	258.2155172	171	220.9807898	222	91.60067633	60	35.256513	32	239.5981535	63.42859468	4.379686071	2.130827463	0.00000000	0.00000000
Cbr3	25.00786549	32.37104151	21	48.16884632	48	11.35545574	7	8.1361184	7	40.26994391	9.745787068	4.329299013	2.114133447	0.00018859	0.01124437
Tns1	66.10197307	127.2257213	84	78.95718107	79	44.66479259	29	13.560197	12	103.0914512	29.11249495	4.28613094	2.099675925	0.00000009	0.00001356
2810025M15Rik	23.83703673	24.84289232	16	50.15519049	50	14.38357727	9	5.9664868	5	37.49904141	10.17503205	4.280358027	2.097731475	0.00028863	0.01599735
Cd209a	45.51103633	65.49489795	43	79.95035316	80	21.9538811	14	14.645013	13	72.72262555	18.2994471	4.197717523	2.069605087	0.00000284	0.00029339
Cdr2	55.41308153	113.6750528	75	67.03911601	67	21.9538811	14	18.984276	17	90.35708438	20.46907868	4.190815301	2.06723094	0.00000082	0.00009771
Fcgrt	242.5505331	428.3516889	284	357.045366	359	85.54443326	56	99.260644	91	392.6985274	92.40253882	4.178127066	2.062856369	0.00000000	0.00000000
Tenm4	50.19574309	95.6074947	63	67.03911601	67	15.89763804	10	22.238724	20	81.32330535	19.06818082	4.078074237	2.027888038	0.00000274	0.00028532
Slc25a10	20.41912853	32.37104151	21	33.27126498	33	6.813273446	4	9.2209342	8	32.82115325	8.017103812	4.054035299	2.019358651	0.00101840	0.04377442
Abcc1	174.6513739	256.7098873	170	297.4550407	299	88.57255479	58	55.868013	51	277.082464	72.22028388	4.045497566	2.016317154	0.00000000	0.00000000
Gypc	83.36244698	151.3157987	100	116.6977205	117	28.01012417	18	37.426145	34	134.0067596	32.71813438	3.977260773	1.991775157	0.00000003	0.00000430
Ece1	26.32779249	53.44985924	35	31.2849208	31	11.35545574	7	9.2209342	8	42.36739002	10.28819496	3.909922926	1.967140169	0.00037236	0.01931651
Adap2	141.5318607	214.5522519	142	235.8783711	237	52.23509642	34	63.461723	58	225.2153115	57.84840994	3.886905921	1.958622189	0.00000000	0.00000002
Dok2	61.27565594	86.57371568	57	106.7659996	107	29.52418493	19	22.238724	20	96.66985762	25.88145427	3.866096634	1.950877698	0.00000058	0.00007155
Dennd2a	25.53247456	38.39356086	25	41.21664169	41	14.38357727	9	8.1361184	7	39.80510128	11.25984783	3.852522156	1.945803254	0.00034664	0.01839800
Trerf1	39.26656435	62.48363827	41	62.07325556	62	11.35545574	7	21.153908	19	62.27844692	16.25468178	3.845612351	1.943213342	0.00003229	0.00250181
Tgfbr2	58.20288332	79.04556649	52	101.8001391	102	34.06636723	22	17.89946	16	90.4228528	25.98291385	3.842093144	1.941892495	0.00000097	0.00011162
Arntl	47.01565922	88.07934551	58	62.07325556	62	18.92575957	12	18.984276	17	75.07630054	18.95501791	3.821037591	1.933964451	0.00000836	0.00074845
Plxnd1	60.76812968	76.03430681	50	115.7045484	116	28.01012417	18	23.323539	21	95.86942759	25.66683178	3.801420217	1.926538512	0.00000085	0.00009945
Igfbp4	95.78824855	152.8214285	101	138.5475064	139	67.37570407	44	24.408355	22	145.6844675	45.89202963	3.781002681	1.918768872	0.00000001	0.00000200
Arf2	37.23468924	47.42733989	31	69.02546018	69	18.92575957	12	13.560197	12	58.22640004	16.24297845	3.742287213	1.903920287	0.00004386	0.00324811
Fpr1	33.75229337	32.37104151	21	51.14836258	51	47.69291412	31	3.7968552	3	41.75970205	25.74488468	3.741334455	1.903552941	0.00017131	0.01034836
Kctd12	179.6012356	274.7774454	182	285.5369756	287	94.62879786	62	63.461723	58	280.1572105	79.04526066	3.689932099	1.883594268	0.00000000	0.00000000
Ado	42.21362118	51.94422941	34	80.94352525	81	15.89763804	10	20.069092	18	66.44387733	17.98336504	3.669929994	1.875752543	0.00002644	0.00210154
Iqck	41.23634435	53.44985924	35	73.99132063	74	9.841394977	6	27.662803	25	63.72058994	18.75209876	3.619163975	1.855656474	0.00005138	0.00371450
Sema4a	36.12824393	73.02304713	48	39.23029751	39	21.9538811	14	10.30575	9	56.12667232	16.12981553	3.607248081	1.850898645	0.00010637	0.00678279
Gas2l1	43.76897387	74.52867697	49	63.06642765	63	17.41169881	11	20.069092	18	68.79755231	18.74039542	3.5963906	1.846549719	0.00002532	0.00203080
Adora2b	30.56105904	23.33726249	15	73.99132063	74	11.35545574	7	13.560197	12	48.66429156	12.45782653	3.579629054	1.839810093	0.00038638	0.01976463
Serpinb6a	1183.42435	1807.50862	1200	1860.707909	1873	639.6906735	422	425.7902	392	1834.108265	532.7404347	3.57601794	1.838353974	0.00000000	0.00000000
Snx5	144.4338656	295.8562631	196	161.3904645	162	46.17885335	30	74.309881	68	228.6233638	60.24436733	3.533871008	1.82124938	0.00000000	0.00000027
Rtn4rl1	46.84228799	54.95548908	36	78.95718107	79	43.15073182	28	10.30575	9	66.95633508	26.72824089	3.511118956	1.811930875	0.00002012	0.00166403
SLC25A10	60.27264443	91.09060519	60	95.84110659	96	26.4960634	17	27.662803	25	93.46585589	27.07943297	3.451538531	1.78723959	0.00000353	0.00036062
Pepd	525.9975927	890.5800491	591	725.512211	730	297.5129405	196	190.38517	175	808.04613	243.9490554	3.402933604	1.766779002	0.00000000	0.00000000
Ptgr1	101.5924018	125.7200915	83	186.2197667	187	53.74915718	35	40.680592	37	155.9699291	47.21487457	3.389895781	1.76124092	0.00000006	0.00000863
Capn2	309.0107061	609.0272694	404	343.1409567	345	182.4443223	120	101.43028	93	476.0841131	141.9372991	3.350130348	1.74421723	0.00000000	0.00000000
Fam213b	44.14123583	56.46111892	37	73.99132063	74	32.55230646	21	13.560197	12	65.22621977	23.05625189	3.314578867	1.728825581	0.00004968	0.00360631
Cttn	64.0242129	128.7313511	85	68.0322881	68	37.09448876	24	22.238724	20	98.38181961	29.66660618	3.247246879	1.699217073	0.00000753	0.00069107
Ctsb	1871.538936	3108.3728	2064	2609.559664	2627	990.9527712	654	777.27051	716	2858.966232	884.1116408	3.246523172	1.698895507	0.00000000	0.00000000
Renbp	45.97644434	80.55119632	53	58.10056721	58	29.52418493	19	15.729829	14	69.32588176	22.62700691	3.245825702	1.698585531	0.00005480	0.00393086
Prkch	46.02219589	44.41608022	29	94.8479345	95	28.01012417	18	16.814645	15	69.63200736	22.41238442	3.238595825	1.695368432	0.00005930	0.00422094
Fam214a	40.26671177	63.98926811	42	53.13470676	53	32.55230646	21	11.390566	10	58.56198743	21.97143611	3.212923046	1.683886427	0.00013317	0.00834660
Ctsc	77.95783947	122.7088318	81	110.7386879	111	50.72103565	33	27.662803	25	116.7237599	39.19191909	3.206046407	1.680795308	0.00000145	0.00016181
Apba1	46.1132342	79.04556649	52	62.07325556	62	18.92575957	12	24.408355	22	70.55941102	21.66705737	3.158998063	1.659467053	0.00007930	0.00525728
Hlcs	29.59609058	39.8991907	26	48.16884632	48	18.92575957	12	11.390566	10	44.03401851	15.15816266	3.134658776	1.648308407	0.00076517	0.03486754
Tspan3	226.7494849	306.395672	203	357.045366	359	170.3318361	112	73.225066	67	331.720519	121.7784508	3.096609919	1.630689658	0.00000000	0.00000006
Ap2a2	92.45360022	157.3383181	104	123.6499251	124	41.63667106	27	47.189487	43	140.4941216	44.41307887	3.085904043	1.625693202	0.00000089	0.00010335
Rasal2	63.28260591	76.03430681	50	114.7113763	115	32.55230646	21	29.832434	27	95.37284154	31.19237028	3.08318508	1.624421495	0.00001245	0.00106354
Slc37a2	38.22405418	62.48363827	41	53.13470676	53	12.86951651	8	24.408355	22	57.80917252	18.63893584	3.080432632	1.623132985	0.00032818	0.01767352
Gpr146	49.69999013	62.48363827	41	86.90255779	87	17.41169881	11	32.002066	29	74.69309803	24.70688224	3.058449808	1.6128006	0.00007718	0.00517265
Cebpd	71.80299022	100.1243842	66	111.73186	112	47.69291412	31	27.662803	25	105.9281221	37.67785833	3.024842076	1.596859823	0.00000662	0.00062001
Nrp2	40.46290684	59.47237859	39	62.07325556	62	15.89763804	10	24.408355	22	60.77281708	20.15299661	3.004712017	1.587226724	0.00027512	0.01540314

Irf4	40.16596822	62.48363827	41	58.10056721	58	18.92575957	12	21.153908	19	60.29210274	20.0398337	2.975506366	1.573135204	0.00029629	0.01629062
Nrros	224.7818133	322.9576002	214	343.1409567	345	137.0224993	90	96.006197	88	333.0492785	116.5143482	2.956772638	1.564023311	0.00000000	0.00000023
Mpp6	615.6938684	737.0058056	489	1098.944916	1106	274.802029	181	352.02272	324	917.975361	313.4123757	2.92304213	1.547470623	0.00000000	0.00000000
Cdk14	39.48378965	51.94422941	34	65.05277183	65	21.9538811	14	18.984276	17	58.49850062	20.46907868	2.915822428	1.543902863	0.00037148	0.01931651
Pip5k1c	33.60823725	47.42733989	31	47.17567423	47	29.52418493	19	10.30575	9	47.30150706	19.91496745	2.913548881	1.542777515	0.00084874	0.03773929
Hpse	67.03119357	101.6300141	67	97.82745077	98	35.58042799	23	33.086881	30	99.72873241	34.33365473	2.91263196	1.542323414	0.00001942	0.00161340
Wwp1	231.1223254	392.2165728	260	294.4755244	296	138.5365601	91	99.260644	91	343.3460486	118.8986022	2.899626886	1.535867271	0.00000000	0.00000035
Ehd4	146.3211781	201.0015834	133	227.9329944	229	96.14285862	63	60.207276	55	214.4672889	78.17506736	2.894078072	1.533103841	0.00000009	0.00001260
Abca1	123.3136446	146.7989092	97	205.0900364	206	100.6850409	66	40.680592	37	175.9444728	70.68281644	2.883536983	1.527839526	0.00000039	0.00005097
Fam126a	156.5954624	244.6648486	162	220.9807898	222	81.00225097	53	79.73396	73	232.8228192	80.3681056	2.880900783	1.526519975	0.00000006	0.00000895
Uaca	85.64968396	140.7763898	93	111.73186	112	53.74915718	35	36.341329	33	126.2541249	45.045243	2.86385634	1.517959124	0.00000552	0.00053056
Emb	114.7945591	267.2492962	177	89.88207405	90	46.17885335	30	55.868013	51	178.5656851	51.02343316	2.856053341	1.514022924	0.00000199	0.00021247
Ccr5	43.6792405	60.97800843	40	68.0322881	68	23.46794187	15	22.238724	20	64.50514826	22.85333274	2.848186875	1.510043808	0.00028964	0.01599735
Aldh7a1	57.41532771	109.1581632	72	63.06642765	63	18.92575957	12	38.51096	35	86.11229545	28.71835998	2.846394235	1.509135494	0.00010732	0.00681915
Cd200r1	34.39153773	32.37104151	21	69.02546018	69	20.43982034	13	15.729829	14	50.69825085	18.08482461	2.821659544	1.496543926	0.00110895	0.04725013
Wfdc17	286.7581027	342.5307881	227	504.0348352	507	149.1349854	98	151.3318	139	423.2828116	150.2333938	2.816890624	1.494103547	0.00000000	0.00000012
Plxnc1	91.34477947	136.2595003	90	133.581646	134	46.17885335	30	49.359118	45	134.9205731	47.7689858	2.812840224	1.492027607	0.00000488	0.00048135
Phlpp2	36.86245691	45.92171005	30	54.12787885	54	37.09448876	24	10.30575	9	50.02479445	23.70011936	2.809308266	1.49021494	0.00082525	0.03696303
Jam2	39.37558177	42.91045038	28	72.99814854	73	20.43982034	13	21.153908	19	57.95429946	20.79686408	2.776803422	1.473425048	0.00066952	0.03177025
Arhgap26	45.85449604	59.47237859	39	74.98449272	75	23.46794187	15	25.493171	23	67.22843566	24.48055642	2.753558159	1.461297081	0.00033924	0.01816258
Vat1	41.19960556	53.44985924	35	63.06642765	63	32.55230646	21	15.729829	14	58.25814345	24.14106768	2.719616842	1.443403409	0.00062381	0.03006708
Ccl24	208.689857	202.5072132	134	408.6903146	411	111.2834663	73	112.27843	103	305.5987639	111.78095	2.662888918	1.412992248	0.00000006	0.00000950
Pltp	177.6308466	246.1704785	163	264.6803617	266	118.8537701	78	80.818776	74	255.4254201	99.83627307	2.659297397	1.411045127	0.00000017	0.00002363
Dnajc10	78.18813627	116.6863124	77	105.7728275	106	58.29133948	38	32.002066	29	111.22957	45.14670258	2.644820235	1.403169668	0.00003565	0.00273616
Eps8	169.1986301	219.0691414	145	271.6325663	273	90.08661556	59	96.006197	88	245.3508539	93.04640629	2.64334154	1.402362844	0.00000029	0.00003788
Arhgap31	123.7598392	181.4283955	120	176.2880458	177	74.9460079	49	62.376908	57	178.8582206	68.66145779	2.635180006	1.397901514	0.00000247	0.00025938
B4galt5	55.44633371	94.10186486	62	67.03911601	67	34.06636723	22	26.577987	24	80.57049044	30.32217699	2.628962078	1.394493332	0.00024832	0.01420578
Tprgl	124.8094589	211.5409922	140	151.4587436	152	74.9460079	49	61.292092	56	181.4998679	68.1190499	2.622376226	1.39087468	0.00000287	0.00029488
Kifc3	49.70600495	62.48363827	41	64.05959974	64	59.80540025	39	12.475382	11	63.271619	36.14039089	2.56360021	1.358171293	0.00065704	0.03142149
Psd3	61.68892566	100.1243842	66	77.96400899	78	35.58042799	23	33.086881	30	89.0441966	34.33365473	2.550059991	1.350531188	0.00021654	0.01258401
Stab1	76.01234532	130.236981	86	80.94352525	81	67.37570407	44	25.493171	23	105.5902531	46.43443752	2.548837922	1.349839637	0.00009145	0.00597621
Plec	281.208037	371.137755	246	430.5401006	433	179.4162007	118	143.73809	132	400.8389278	161.5771462	2.530450686	1.339394359	0.00000002	0.00000373
Stard8	84.7187005	137.7651302	91	102.7933112	103	59.80540025	39	38.51096	35	120.2792207	49.15818032	2.499051139	1.321380424	0.00006187	0.00433640
Frmd4b	138.1558462	246.1704785	163	152.4519157	153	84.0303725	55	69.970618	64	199.3111971	77.00049533	2.487996712	1.314984579	0.00000521	0.00050851
Ulk2	56.77150667	91.09060519	60	71.01180436	71	34.06636723	22	30.91725	28	81.05120478	32.49180856	2.457537537	1.297213453	0.00051085	0.02508348
Slc9a3r2	287.185882	342.5307881	227	470.2669841	473	182.4443223	120	153.50143	141	406.3988861	167.972878	2.453116904	1.294615988	0.00000005	0.00000815
Sat1	166.2253502	235.6310696	156	236.8715432	238	85.54443326	56	106.85435	98	236.2513064	96.19939407	2.447052924	1.291045304	0.00000224	0.00023615
Cd86	105.5440096	127.2257213	84	172.3153574	173	53.74915718	35	68.885802	63	149.7705394	61.31747979	2.441412459	1.287716048	0.00003000	0.00235441
Tcta	77.52707568	146.7989092	97	77.96400899	78	44.66479259	29	40.680592	37	112.3814591	42.67269227	2.436937908	1.285069492	0.00016850	0.01021261
Hmox1	106.5360173	178.4171358	118	126.6294413	127	59.80540025	39	61.292092	56	152.5232886	60.54874607	2.435984182	1.284504765	0.00003114	0.00242331
Emp3	240.9414862	353.070197	234	331.2228917	333	132.480317	87	146.99254	135	342.1465443	139.736428	2.432168117	1.282242955	0.00000024	0.00003174
Snx6	128.6040487	188.9565446	125	169.3358412	170	99.17098015	65	56.952829	52	179.1461929	78.06190445	2.429916252	1.280906592	0.00001132	0.00098542
Anxa2	1254.278593	1949.037825	1294	1615.394403	1626	735.0765018	485	717.60564	661	1782.216114	726.341072	2.421953727	1.276171301	0.00000000	0.00000000
Snx13	96.19573725	152.8214285	101	118.6840646	119	62.83352178	41	50.443934	46	135.7527466	56.6387279	2.387154628	1.25529202	0.00007088	0.00483904
Gltp	156.1188597	208.5297325	138	227.9329944	229	100.6850409	66	87.327671	80	218.2313635	94.00635583	2.350362571	1.232883326	0.00000783	0.00071286
Atp6ap1	70.08854922	97.11312454	64	96.83427868	97	52.23509642	34	34.171697	31	96.97370161	43.20339683	2.349990723	1.232655061	0.00036029	0.01892232
Dse	70.4109787	97.11312454	64	97.82745077	98	26.4960634	17	60.207276	55	97.47028765	43.35166975	2.33005271	1.220362591	0.00048041	0.02384381
Myo1e	65.74411044	48.93296973	32	134.5748181	135	50.72103565	33	28.747618	26	91.75389389	39.73432699	2.3272125	1.21860295	0.00069992	0.03262287
Sestd1	109.3476995	164.8664672	109	139.5406785	140	74.9460079	49	58.037645	53	152.2035729	66.49182622	2.30870519	1.20708396	0.00006898	0.00474439
Prr5l	81.68887922	92.59623503	61	132.5884739	133	59.80540025	39	41.765408	38	112.5923545	50.785404	2.304163087	1.204242833	0.00025765	0.01463661
Arhgef3	77.85203733	149.8101689	99	72.99814854	73	44.66479259	29	43.935039	40	111.4041587	44.29991595	2.271331143	1.183538056	0.00049174	0.02427493
Gas7	141.0303224	194.979064	129	189.199283	190	114.3115878	75	65.631355	60	192.0891735	89.97147142	2.271154924	1.183426121	0.00002862	0.00225576
Setd3	81.90832289	113.6750528	75	111.73186	112	58.29133948	38	43.935039	40	112.7034564	51.1131894	2.258318566	1.175249012	0.00034181	0.01824732
Lyz1	3002.714955	4105.099753	2726	4190.689629	4219	1961.465722	1295	1753.6047	1616	4147.894691	1857.53522	2.249266914	1.169454871	0.00000000	0.00000000
Dab2	97.1319031	197.9903237	131	80.94352525	81	61.31946101	40	48.274302	44	139.4669245	54.79688174	2.246010274	1.167364527	0.00026316	0.01486955
Nrp1	719.4187424	1057.704961	702	910.2422196	916	560.9595137	370	348.76828	321	983.9735903	454.8638944	2.243237448	1.165582339	0.00000000	0.00000018
Leprot	88.73867306	124.2144616	82	120.6704088	121	47.69291412	31	62.376908	57	122.4424352	55.0349109	2.21035507	1.144278142	0.00036100	0.01892232
Parl	78.36225351	79.04556649	52	137.5543343	138	43.15073182	28	53.698381	49	108.2999504	48.42455661	2.207208477	1.142222903	0.00062622	0.03010432
Mfsd6	179.3704772	201.0015834	133	292.4891802	294	112.797527	74	111.19362	102	246.7453818	111.9955725	2.206814758	1.141965534	0.00001677	0.00140622
Anxa7	98.28199899	131.7426108	87	138.5475064	139	58.29133948	38	64.546539	59	135.1450586	61.41893936	2.197185888	1.135656931	0.00025824	0.01463661

Armc8	69.57311065	79.04556649	52	111.73186	112	44.66479259	29	42.850224	39	95.38871325	43.75750806	2.195099566	1.13428638	0.00098022	0.04255492
Naaa	97.63842244	116.6863124	77	151.4587436	152	56.77727871	37	65.631355	60	134.072528	61.20431687	2.192076133	1.132297906	0.00027882	0.01556326
Evi2a	470.656691	675.2749823	448	620.2359696	624	276.3160897	182	310.79972	286	647.7554759	293.5579062	2.188968024	1.13025088	0.00000007	0.00001045
Arhgap17	76.20305028	97.11312454	64	111.73186	112	47.69291412	31	48.274302	44	104.4224923	47.98360829	2.186290731	1.128485262	0.00073445	0.03384961
P2rx4	75.86750964	104.6412737	69	101.8001391	102	55.26321795	36	41.765408	38	103.2207064	48.51431285	2.177457643	1.122644655	0.00078909	0.03569155
Mtss1	237.465099	353.070197	234	297.4550407	299	164.2755931	108	135.05957	124	325.2626188	149.6675792	2.175234239	1.121170765	0.00000565	0.00054030
Mef2c	186.5139286	199.4959535	132	312.352622	314	106.741284	70	127.46585	117	255.9242877	117.1035694	2.170918207	1.118305371	0.00002101	0.00171417
Lamp1	818.7821568	1163.09905	772	1081.067819	1088	506.4533261	334	524.50843	483	1122.083434	515.4808792	2.164695113	1.114163843	0.00000000	0.00000026
Scamp2	185.5178221	277.7887051	184	229.9193386	231	126.4240739	83	107.93917	99	253.8540218	117.1816223	2.158229996	1.109848617	0.00002362	0.00190222
Oxct1	232.8863589	313.9238212	208	321.2911708	323	153.6771677	101	142.65328	131	317.607496	148.1652218	2.15409805	1.10708392	0.00000784	0.00071286
Snx2	1222.034155	1675.013195	1112	1639.230533	1650	898.5950644	593	675.29783	622	1657.121864	786.9464455	2.149080577	1.103719576	0.00000000	0.00000001
March1	207.969434	253.6986277	168	306.3935895	308	162.7615323	107	109.02399	100	280.0461086	135.8927594	2.148951539	1.103632949	0.00001487	0.00125245
Atp6v1c1	82.54190295	118.1919423	78	107.7591717	108	46.17885335	30	58.037645	53	112.975557	52.10824894	2.143195716	1.099763602	0.00076031	0.03481890
Ap2m1	91.68891486	131.7426108	87	118.6840646	119	58.29133948	38	58.037645	53	125.2133377	58.16449201	2.138343022	1.096493301	0.00052607	0.02576187
Cbfb	74.23943642	86.57371568	57	115.7045484	116	43.15073182	28	51.52875	47	101.139132	47.33974082	2.136269577	1.095093713	0.00114635	0.04842547
Akt2	121.1220365	148.304539	98	181.2539062	182	64.34758254	42	90.582118	83	164.7792226	77.46485032	2.134922649	1.0941838	0.00019174	0.01137772
Ctsl	1140.99406	1220.312984	810	1891.496243	1904	691.1687395	456	760.99827	701	1555.904614	726.0835066	2.133971291	1.093540767	0.00000000	0.00000003
Hsd17b4	99.25995151	131.7426108	87	135.5679901	136	74.9460079	49	54.783197	50	133.6553005	64.86460254	2.121348715	1.084981796	0.00043959	0.02211672
Fdft1	110.4000794	149.8101689	99	149.4723994	150	73.43194714	48	68.885802	63	149.6412841	71.15887476	2.110201673	1.077380884	0.00032387	0.01749291
Usp24	177.7410186	226.5972906	150	254.7486408	256	117.3397093	77	112.27843	103	240.6729657	114.8090716	2.109395896	1.076829889	0.00004685	0.00344156
Kank3	81.14448099	121.2032019	80	99.81379494	100	47.69291412	31	55.868013	51	110.5084984	51.78046354	2.090948546	1.06415756	0.00116374	0.04878466
Klf6	176.9713247	283.8112244	188	195.1583155	196	133.9943778	88	94.921381	87	239.48477	114.4578795	2.07982715	1.056463634	0.00007042	0.00482524
Rac1	207.6448817	222.0804011	147	337.1819242	339	103.7131625	68	167.60404	154	279.6311626	135.6586007	2.075407399	1.053394563	0.00003793	0.00287866
Klf4	207.1543043	345.5420478	229	221.9739619	223	109.7694055	72	151.3318	139	283.7580048	130.5506038	2.073749621	1.052241717	0.00004033	0.00303543
Bmp2k	141.8803654	208.5297325	138	175.2948737	176	93.11473709	61	90.582118	83	191.9123031	91.8484276	2.066368317	1.047097428	0.00018663	0.01116369
Uap1l1	141.8744037	184.4396551	122	197.1446597	198	77.97412943	51	107.93917	99	190.7921574	92.95665005	2.05496064	1.039110761	0.00021025	0.01233417
Lmna	153.9704692	202.5072132	134	203.1036922	204	129.4521955	85	80.818776	74	202.8054527	105.1354857	2.025069254	1.017971247	0.00020128	0.01188523
Serinc3	374.9923601	532.2401477	353	468.28064	471	280.858272	185	218.59038	201	500.2603938	249.7243264	2.022198653	1.015924729	0.00000394	0.00039741
Clic4	128.4675807	173.9002463	115	170.3290133	171	77.97412943	51	91.666934	84	172.1146298	84.82053166	2.018465519	1.013258942	0.00041494	0.02099148
Tlr4	110.7361152	133.2482406	88	162.3836365	163	71.91788637	47	75.394697	69	147.8159386	73.65629174	2.013721829	1.009864406	0.00071297	0.03314661
S100a4	136.8201713	181.4283955	120	183.2402504	184	93.11473709	61	89.497302	82	182.3343229	91.3060197	2.001917387	1.00138244	0.00038029	0.01958136
Larp1b	101.2544592	136.2595003	90	133.581646	134	67.37570407	44	67.800987	62	134.9205731	67.58834534	1.99342524	0.995249501	0.00112239	0.04771200
Lpl	576.9402546	560.8471146	372	964.8666845	971	454.9752601	300	327.07196	301	762.8568995	391.0236097	1.984470307	0.988753976	0.00000070	0.00008406
C5ar1	106.6381126	133.2482406	88	149.4723994	150	74.9460079	49	68.885802	63	141.36032	71.91590515	1.983958762	0.988382039	0.00102023	0.04377442
Ptpra	153.0901224	176.9115059	117	227.9329944	229	108.2553447	71	99.260644	91	202.4222502	103.7579946	1.96934732	0.977717572	0.00035087	0.01856882
Glud1	654.9833516	988.4459885	656	749.3483411	754	501.9111438	331	380.22793	350	868.8971648	441.0695383	1.964422486	0.974105242	0.00000044	0.00005574
Eps15	115.3584356	204.012843	135	107.7591717	108	84.0303725	55	65.631355	60	155.8860073	74.83086376	1.954312758	0.966661367	0.00116622	0.04878466
Itm2b	333.4900788	437.3854679	290	444.4445098	447	217.2677199	143	234.86262	216	440.9149889	226.0651687	1.947304052	0.961478164	0.00002053	0.00168323
Trim26	114.2106751	161.8552076	107	140.5338506	141	77.97412943	51	76.479513	70	151.1945291	77.22682116	1.940566063	0.956477548	0.00119657	0.04960220
Metrnl	406.4919001	410.2841308	272	660.9560252	665	295.9988797	195	258.72856	238	535.620078	277.3637223	1.936571823	0.953505009	0.00001004	0.00089065
Picalm	340.3915136	390.7109429	259	505.0280072	508	208.1833553	137	257.64375	237	447.8694751	232.9135522	1.925921322	0.945548768	0.00002598	0.00207411
Abcg1	173.6867827	194.979064	129	240.8442316	242	182.4443223	120	76.479513	70	217.9116478	129.4619176	1.925483146	0.945220495	0.00038067	0.01958136
Nsf	303.9786994	405.7672413	269	393.7927333	396	209.6974161	138	206.65741	190	399.7799873	208.1774116	1.918522434	0.939995635	0.00004563	0.00336531
Tnfrsf11a	144.8290416	199.4959535	132	182.2470783	183	96.14285862	63	101.43028	93	190.8715159	98.78656729	1.917562752	0.93927379	0.00069811	0.03262123
Rbpj	346.749311	384.6884236	255	502.048491	505	299.0270012	197	201.23333	185	443.3684573	250.1301647	1.843700039	0.882603955	0.00007709	0.00517265
Aplp2	180.8300774	237.1366995	157	226.9398223	228	149.1349854	98	110.1088	101	232.0382609	129.6218938	1.830143442	0.871956728	0.00083524	0.03731951
Abca9	175.8829998	228.1029204	151	226.9398223	228	118.8537701	78	129.63549	119	227.5213714	124.2446282	1.825626311	0.868391489	0.00095006	0.04144172
Adam9	397.6230581	508.1500703	337	516.9460723	520	283.8863936	187	281.5097	259	512.5480713	282.698045	1.815136681	0.860078188	0.00006636	0.00459877
Neat1	719.014863	1020.064215	677	844.6928617	850	479.2002323	316	532.10214	490	932.3785384	505.6511876	1.813577591	0.85883847	0.00000470	0.00046694
Camk1	291.210953	363.6096058	241	381.8746682	384	220.2958414	145	199.0637	183	372.742137	209.679769	1.79093253	0.840710987	0.00029008	0.01599735
Tgfbi	1043.852442	1093.840077	726	1499.193268	1509	1031.832412	681	550.54401	507	1296.516673	791.1882115	1.769914241	0.823679458	0.00000164	0.00018051
Sec14l1	249.1032885	286.8224841	190	349.0999893	351	173.3599577	114	187.13072	172	317.9612367	180.2453403	1.768358002	0.822410376	0.00063793	0.03058745
Cers5	249.1923762	338.0138986	224	300.4345569	302	173.3599577	114	184.96109	170	319.2242278	179.1605246	1.76471819	0.819437816	0.00066836	0.03177025
Rnf130	389.8317111	517.1838493	343	475.2328446	478	285.4004543	188	281.5097	259	496.2083469	283.4550754	1.742747742	0.801363758	0.00021090	0.01233417
Rbms1	521.014315	666.2412032	442	655.9901648	660	398.9550118	263	362.87088	334	661.115684	380.912946	1.742652123	0.8012846	0.00007180	0.00488373
St6gal1	424.9031016	517.1838493	343	556.6729559	560	339.9066419	224	285.84896	263	536.9284026	312.8778007	1.739420482	0.798606727	0.00016271	0.00992700
Serinc1	268.4762129	380.1715341	252	303.4140732	305	209.6974161	138	180.62183	166	341.7928036	195.1596222	1.736348554	0.796056583	0.00076447	0.03486754
Twf1	438.8178357	554.8245952	368	551.7070954	555	344.4488242	227	304.29083	280	553.2658453	324.369826	1.716813089	0.77973298	0.00020547	0.01209351
Fam102b	268.3686343	390.7109429	259	293.4823523	295	194.5568084	128	194.72443	179	342.0966476	194.640621	1.715469708	0.778603651	0.00099958	0.04322020
Rap1a	339.7943307	338.0138986	224	520.9187607	524	233.9223883	154	266.32228	245	429.4663296	250.1223318	1.708361464	0.77261326	0.00055519	0.02690112

Cyp27a1	292.3647293	365.1152357	242	371.9429473	374	212.7255376	140	219.6752	202	368.5290915	216.2003671	1.704081023	0.768993932	0.00088198	0.03902828
Pitpna	534.8642486	669.2524629	444	677.8399507	682	345.962885	228	446.4017	411	673.5462068	396.1822904	1.69787712	0.763732051	0.00014011	0.00872208
Actg1	1110.373817	1425.078642	946	1375.046757	1384	757.7874132	500	883.58246	814	1400.062699	820.6849353	1.697226092	0.763178763	0.00000580	0.00054927
Fam63b	321.7748525	393.7222026	261	414.6493472	417	246.0348744	162	232.69299	214	404.1857749	239.3639302	1.696703752	0.762734689	0.00073546	0.03384961
Atp6ap2	433.4621179	575.903413	382	506.0211793	509	367.1597357	242	284.76414	262	540.9622962	325.9619397	1.675596504	0.744674779	0.00040655	0.02068099
Klc1	383.1161194	453.9473961	301	501.0553189	504	311.1394874	205	266.32228	245	477.5013575	288.7308814	1.675050224	0.744204353	0.00059291	0.02865303
Rab11fip5	364.2832749	363.6096058	241	542.7685466	546	247.5489352	163	303.20601	279	453.1890762	275.3774736	1.640495899	0.714131987	0.00115204	0.04842547
Samhd1	425.2205748	431.3629485	286	611.2974208	615	224.8380237	148	433.38391	399	521.3301847	329.110965	1.630777377	0.705559848	0.00090929	0.04004354
C3ar1	452.3828235	636.1286065	422	489.1372538	492	362.6175534	239	321.64788	296	562.6329302	342.1327169	1.619970235	0.695967306	0.00083762	0.03733489
Clec4a3	491.7624034	532.2401477	353	676.8467786	681	385.3284649	254	372.63422	343	604.5434632	378.9813436	1.604942438	0.682521555	0.00081859	0.03675429
Iqgap2	508.2913893	566.8696339	376	666.9150578	671	462.5455639	305	336.8353	310	616.8923458	399.6904327	1.58994693	0.668978611	0.00094601	0.04136297
Cndp2	978.1075241	1248.91995	829	1144.630833	1152	727.5061979	480	791.37312	729	1196.775392	759.4396568	1.563629151	0.644898387	0.00019207	0.01137772
Rab11a	870.4249559	1041.143033	691	1073.122442	1080	729.0202587	481	638.41409	588	1057.132738	683.7171743	1.559913516	0.641466046	0.00030703	0.01678083
Rin2	1363.857291	1477.775686	981	1805.090272	1817	1227.146251	810	945.41696	871	1641.432979	1086.281604	1.548034402	0.630437532	0.00007749	0.00517497
Adam10	646.1012819	717.4326177	476	850.6518942	856	470.1158678	310	546.20475	503	784.042256	508.1603079	1.545596972	0.628164173	0.00100025	0.04322020
Ahnak	2545.579113	3022.551899	2007	3102.17302	3123	2262.763814	1494	1794.8277	1654	3062.36246	2028.795766	1.536233673	0.619397678	0.00000571	0.00054377

Supplementary Table 3: List of antibodies used for immunophenotyping.

Antibody	Company	Catalogue number
Annexin V FITC	BD Biosciences	51-65874X
CD102 (ICAM2) eFluor 450	ebioscience	48-1021
CD11b Alexa Fluor 647	BD Biosciences	557686
CD11b Biotin	BD Biosciences	51-017125
CD115 PE	TONBO biosciences	50-1152
CD11c FITC	ebioscience	11-0114
CD11b PE-Cy7	BD Biosciences	552850
CD115 Biotin	ebioscience	13-1152
CD16/CD32 (Mouse BD Fc Block)	BD Biosciences	553142
CD3e Alexa Fluor 488	ebioscience	53-0031
CD45 APC-Cy7	BioLegend	103116
CD45 eFluor 450	ebioscience	48-0451
CD45 PerCP-Cy5.5	BD Biosciences	550994
CD45.1 V450	BD Biosciences	560520
CD45.2 Biotin	BD Biosciences	553771
CD45.2 PerCP-Cy5.5	invitrogen	45-0454-82
CD45R/B220 PerCP-Cy5.5	BD Biosciences	561101
F4/80 PE	BioLegend	123110
F4/80 PE-Cy7	BioLegend	123114
GATA-6 (D61E4)	Cell signaling Technology	5851
Goat anti rabbit Alexa Fluor 647	invitrogen	A-21245
ki-67 APC	Biolegend	652405
Ly-6C APC	BD Biosciences	560595
Ly-6C APC-Cy7	BD Biosciences	560596
Ly-6C FITC	BD Biosciences	553104
Ly-6G PerCP-Cy5.5	BioLegend	127615
MHC class II (I-A/I-E) Brilliant Violet 605	BioLegend	107639
MHC class II (I-A/) FITC	ebioscience	Nov-22
Siglec-F PE	BD Biosciences	552126
Streptavidin Pacific Blue	life technologies	S11222
Streptavidin Alexa Fluor 488	life technologies	S11223
Tim-4 Alexa Fluor 647	BioLegend	130008
Tim-4 PE	BioLegend	130006

Supplementary Table 4: Gating strategies for flow cytometry.

Cell type	Gating strategy
TRMs	$CD45^{+}$ $B220^{-}$ $CD11b^{+}$ $Cd115^{+}$ $F480^{hi}$ $MHCII^{lo}$
BDMs	$CD45^{+}$ $B220^{-}$ $CD11b^{+}$ $Cd115^{+}$ $F480^{lo}$ $MHCII^{hi}$
Eosinophils	$CD45^{+}$ $F4/80^{+}$ $CD11b^{+}$ $TIM4^{-}$ $MHCII^{-}$
FL monocytes	$CD11b^{+}$ $Ly6C^{+}$ $F4/80^{int}$
FL macrophages	$CD11b^{int}$ $Ly6C^{-}$ $F4/80^{+}$
YS macrophages	$CD11b^{+}$ $Ly6C^{-}$ $F4/80^{hi}$
Kupffer cells	$CD45^{+}$ $MHCII^{+}$ $F4/80^{hi}$ $CD11b^{lo}$ $Ly6C^{lo}$
Microglia	$CD45^{LO}$ $CD11b^{+}$
PB Monocytes Ly6Chi	$CD45^{+}$ $F4/80^{-}$ $CD11b^{+}$ $CD115^{+}$ $Ly6C^{hi}$
PB Monocytes Ly6Clo	$CD45^{+}$ $F4/80^{-}$ $CD11b^{+}$ $CD115^{+}$ $Ly6C^{lo}$
Small intestine macrophages	$CD45^{+}$ $CD11c^{-}$ $SiglecF^{-}$ $Ly6G^{-}$ $F480^{+}$ $CD11b^{+}$
Splenic red pulp macrophages	$CD45^{+}$ $F4/80^{+}$ MHCII+
Alveolar macrophages	$CD45^{+}$ $CD11b^{+}$ $Ly6G^{-}$ $Siglec\text{-}F^{hi}$ $CD11c^{hi}$
Peritoneal macrophages in tumor ovaries	$CD45^{+}$ $Ly6G^{-}$ $CD11b^{+}$ $F4/80^{+}$ $GATA\text{-}6^{+}$

Supplementary Table 5: Primers used for qPCR amplification.

	Primer name	Sequence (5'-3')
PCR Rxra (genotyping) Rxra	P1	ACCAAGCACATCTGTGCTATCT
	P2	CAACTGTATACCCCATAGTGTT
PCR Rxrb (genotyping) Rxrb	XO141	TCCTCCACTGCACACAGCCC
	WS55	CCGGGAGGGCTGACTTTCATC
Cre transgene amplification	Cre1	AGGTGTAGAGAAGGCACTTAGC
	Cre2	CTAATCGCCATCTTCCAGCAGG
qPCR (gene expression)	*Abca1* -F	AGAGCAAAAAGCGACTCCAC
	Abca1 -R	GGTTTTTGTGCAGTGACAGG
	Abca9 -F	TCTTGGGATCGCCATTTTAG
	Abca9 -R	GCCTGCTCTCCTCTCTTTCA
	Abcc1 -F	CTGAGTTCCTGCGCACCTAT
	Abcc1 -R	CAGGTGCTTTCCTACGGTGT
	Abcg1 -F	TCACCCAGTTCTGCATCCTCTT
	Abcg1 -R	GCAGATGTGTCAGGACCGAGT
	Ap2a2 -F	GAGGCCTAGCGGTCTTCATC
	Ap2a2 -R	AATTTGGATCTAATATTTGCCAGTTC
	Arf2 -F	CGGGAAGAATTGACCAGAAT
	Arf2 -R	CGAAGGGAGTGTAAGCCAAG
	Capn2 -F	GCAGATTCTGAGGCCGTTAC
	Capn2 -R	CCTGATGCGGATCAATTTCT
	Ccnd 1-F	CCCAACAACTTCCTCTCCTG
	Ccnd 1-R	TCCAGAAGGGCTTCAATCTG
	Ccr2 -F	ACACCCTGTTTCGCTGTAGG
	Ccr2 -R	CCTGGAAGGTGGTCAAGAAG
	Cd5l -F	GGAAGACACGTTGGCTCAAT
	Cd5l -R	AGACGCACATCCTCTGGAAT
	Ctsb -F	TGAAGAAGCTGTGTGGCACT
	Ctsb -R	ATTGTTCCCGTGCATCAAA
	Cyp26a1 -F	ATTGAGCACTCGTGGGAGAG
	Cyp26a1 -R	CGCAGCACTGGCTGTAGTT
	Emr1 -F	TGCATCTAGCAATGGACAGC
	Emr1 -R	GCCTTCTGGATCCATTTGAA
	Fcrls -F	CTCCTGTCGGAGTCCAGTC
	Fcrls -R	CACCATTATTTTTCCCTGTGC
	Gata6-F	CTACACAAGCGACCACCTCA
	Gata6-R	TGTAGAGGCCGTCTTGACCT
	Irf4 -F	GTGTGGGAGAACGAGGAGAA
	Irf4 -R	GCCTTTAAACAATGCCCAAG
	Klf4 -F	AAAAGAACAGCCACCCACAC
	Klf4 -R	CGTCCCAGTCACAGTGGTAA
	Klf6 -F	AAAGCTCCCACTTGAAAGCA
	Klf6 -R	GCTTTCGGAAGTGTCTGGTC
	Lamp1 -F	CTATCCAGGCCTACCTGTCG
	Lamp1 -R	GGATACAGTGGGGTTTGTGG
	Mef2c -F	ACTGGGAAACCCCAATCTTC
	Mef2c -R	AGATCTCCGCCCATCAGAC
	Mrc1 -F	ATTGTGGAGCAGATGGAAGG
	Mrc1 -R	TGAATGGAAATGCACAGACG
	Naip1 -F	GCCAAGTGGTTCCCCAAAT
	Naip1 -R	CCCGTTACATGAACAAATCCCT
	Retnla -F	TCGTGGAGAATAAGGTCAAGG
	Retnla -R	GGAGGCCCATCTGTTCATAG
	Rxra-flox/out -F	TACAGTTGTGAGGGCTGCAA
	Rxra-flox/out -R	TTCCGCTGTCTCTTGTCGAT
	Rxrb-flox/out -F	ATGTGAAGCCACCGGTCTTA
	Rxrb-flox/out -R	GCTTGAAGAAACCCTTGCAG
	Tbc1d14 -F	AACTTGCCGAAATTGTTTGC
	Tbc1d14 -R	TCGACAGAATACGTCCCAGA
	Timd4 -F	GGAGCTCTGGTGGCTTTATC
	Timd4 -R	GGGACCACGAGAGGTAATGA
ATAC-seq library preparation	Ad1_noMX	AATGATACGGCGACCACCGAGATCTACACTCGTCGGCAGCGTCAGATGTG
	Ad2.1_TAAGGCGA	CAAGCAGAAGACGGCATACGAGATTCGCCTTAGTCTCGTGGGCTCGGAGATGT
	Ad2.2_CGTACTAG	CAAGCAGAAGACGGCATACGAGATCTAGTACGGTCTCGTGGGCTCGGAGATGT
	Ad2.3_AGGCAGAA	CAAGCAGAAGACGGCATACGAGATTTCTGCCTGTCTCGTGGGCTCGGAGATGT
	Ad2.4_TCCTGAGC	CAAGCAGAAGACGGCATACGAGATGCTCAGGAGTCTCGTGGGCTCGGAGATGT
