# Supplementary material for: RXRs control serous macrophage neonatal expansion and identity and contribute to ovarian cancer progression
Source: Nat Commun. 2020 Apr 3;11:1655. doi: 10.1038/s41467-020-15371-0 (PMC7125161; doi:10.1038/s41467-020-15371-0)
Supplement: Supplementary file 3 — Reporting Summary [file 41467_2020_15371_MOESM3_ESM.pdf]

nature research

Corresponding author(s): Miriam Merad & Mercedes Ricote

Last updated by author(s): Feb 24, 2020

Reporting Summary

Nature Research wishes to improve the reproducibility of the work that we publish. This form provides structure for consistency and transparency in reporting. For further information on Nature Research policies, see Authors & Referees and the Editorial Policy Checklist.

Statistics

For all statistical analyses, confirm that the following items are present in the figure legend, table legend, main text, or Methods section.

n/a	Confirmed	
☐	☒	The exact sample size (*n*) for each experimental group/condition, given as a discrete number and unit of measurement
☐	☒	A statement on whether measurements were taken from distinct samples or whether the same sample was measured repeatedly
☐	☒	The statistical test(s) used AND whether they are one- or two-sided<br>*Only common tests should be described solely by name; describe more complex techniques in the Methods section.*
☒	☐	A description of all covariates tested
☐	☒	A description of any assumptions or corrections, such as tests of normality and adjustment for multiple comparisons
☐	☒	A full description of the statistical parameters including central tendency (e.g. means) or other basic estimates (e.g. regression coefficient) AND variation (e.g. standard deviation) or associated estimates of uncertainty (e.g. confidence intervals)
☐	☒	For null hypothesis testing, the test statistic (e.g. *F*, *t*, *r*) with confidence intervals, effect sizes, degrees of freedom and *P* value noted<br>*Give P values as exact values whenever suitable.*
☒	☐	For Bayesian analysis, information on the choice of priors and Markov chain Monte Carlo settings
☒	☐	For hierarchical and complex designs, identification of the appropriate level for tests and full reporting of outcomes
☐	☒	Estimates of effect sizes (e.g. Cohen's *d*, Pearson's *r*), indicating how they were calculated

Our web collection on statistics for biologists contains articles on many of the points above.

Software and code

Policy information about availability of computer code

Data collection

We have not used any previously unreported custom computer code or algorithm to generate results.

Data analysis

For qPCR analysis we used the commercial softwares qbasePLUS v1.5, and SDS v2.4
For flow cytometry data collection and analysis we used the commercial softwares SP6800 v1.6.3, FACS Diva v6.1, and FlowJo v10.4.2
For image analysis we used the commercial softwares Imaje J v1.46r, and NDP.view 2
For t-SNE analysis we used AP-workflow (Jiménez-Carretero et al., J Immunol, 2018)
For RNA-seq analysis we used the open source and commercial softwares Kallisto v0.43.0, tximport, limma, Panther, GSEA, and Genesis v1.7.6
For ATAC-seq analysis the following open source and commercial software were used: Cutadapt v1.7.1, Bowtie2 v4.1.2, PICARD tools v1.97, samtools v0.1.18, MACS2 v2.1.1, bedtools v2.24.0, DiffBind R package v2.6.6, EdgeR v3.20.9, and HOMER v4.10.3
For statistical analysis and graph creation we used GraphPad PRISM v7.03

For manuscripts utilizing custom algorithms or software that are central to the research but not yet described in published literature, software must be made available to editors/reviewers. We strongly encourage code deposition in a community repository (e.g. GitHub). See the Nature Research guidelines for submitting code & software for further information.

Data

Policy information about availability of data

All manuscripts must include a data availability statement. This statement should provide the following information, where applicable:

- Accession codes, unique identifiers, or web links for publicly available datasets
- A list of figures that have associated raw data
- A description of any restrictions on data availability

All data is available in the main text or the supplementary materials. ULI RNA Seq and ATAC-Seq data are deposited in GEO; accession number GSE129414 and GSE129095, respectively.

Field-specific reporting

Please select the one below that is the best fit for your research. If you are not sure, read the appropriate sections before making your selection.

☒ Life sciences ☐ Behavioural & social sciences ☐ Ecological, evolutionary & environmental sciences

For a reference copy of the document with all sections, see nature.com/documents/nr-reporting-summary-flat.pdf

Life sciences study design

All studies must disclose on these points even when the disclosure is negative.

Sample size	Due to experimental limitations by the reduced number of animals obtained per littler, no sample-size calculation was performed at the moment of experimental design. We used all the animals obtained in each litter and repeated the experiments several times making sure that the data were successfully replicated.
Data exclusions	Animals with any signs of illness were excluded from our studies.<br>Significant outliers were determined using a Grubbs' test (GraphPad) and excluded as considered technical artifacts.<br>In RNA-seq and ATAC-seq experiments we excluded samples that do not randomly clusterized with their experimental group after diagnostic plot analysis.
Replication	All experiments were performed several times, and all attempts at replication were successful.
Randomization	All experimental groups used in our studies contained all mice per litter, independently of their gender or genotype.
Blinding	Bias was avoided by blinded experiments. Organ collection, processing and data collection was always performed blinded. In flow cytometry and qPCR studies embryos and neonate mice were genotyped after the experiments were carried out, and in adult mice the genotype was generally checked at the moment of graph generation. Microscopy studies, ATAC-seq and RNA-seq were not blinded.

Reporting for specific materials, systems and methods

We require information from authors about some types of materials, experimental systems and methods used in many studies. Here, indicate whether each material, system or method listed is relevant to your study. If you are not sure if a list item applies to your research, read the appropriate section before selecting a response.

Materials & experimental systems

n/a	Involved in the study	
☐	☒	Antibodies
☐	☒	Eukaryotic cell lines
☒	☐	Palaeontology
☐	☒	Animals and other organisms
☒	☐	Human research participants
☒	☐	Clinical data

Methods

n/a	Involved in the study	
☒	☐	ChIP-seq
☐	☒	Flow cytometry
☒	☐	MRI-based neuroimaging

Antibodies

Antibodies used	The antibody suppliers, clones and details can be found in Methods.
Validation	According to manufacturer' s data sheets, all the antibodies used in this study had been reported to work in mice for flow cytometry or immunofluorescence. In general, a 1:100 dilution was used, with the exception of anti-ki67 and anti-GATA-6 that were titrated to determine the optimal antibody concentration.

Eukaryotic cell lines

Policy information about cell lines

Cell line source(s)	Upk10 cells were generated by Scarlett U.K. et al. (JEM, 2009).
Authentication	The Upk10 cell line have not been authenticated by our lab independently of the author's authentication.
Mycoplasma contamination	Upk10 cells were tested mycoplasma negative.
Commonly misidentified lines<br>(See ICLAC register)	No commonly misidentified cell lines were used.

Animals and other organisms

Policy information about studies involving animals; ARRIVE guidelines recommended for reporting animal research

Laboratory animals	All the animals used in this study were on the C57BL/6 background. Male and female mice were studied from E13.5 to 70 days of age.
Wild animals	The study did no involved wild animals.
Field-collected samples	The study did not involved samples collected from the field.
Ethics oversight	All experiments were carried out according to local ethical guidelines, and were approved by the Animal Subjects Committee of the Instituto de Salud Carlos III (Madrid, Spain) in accordance with EU Directive 86/609/EEC, or by IACUC at Icahn School of Medicine at Mount Sinai in accordance with NIH guidelines.

Note that full information on the approval of the study protocol must also be provided in the manuscript.

Flow Cytometry

Plots

Confirm that:

- [x] The axis labels state the marker and fluorochrome used (e.g. CD4-FITC).
- [x] The axis scales are clearly visible. Include numbers along axes only for bottom left plot of group (a 'group' is an analysis of identical markers).
- [x] All plots are contour plots with outliers or pseudocolor plots.
- [x] A numerical value for number of cells or percentage (with statistics) is provided.

Methodology

Sample preparation	The sample preparation details can be found in Methods.
Instrument	BD FACSCanto II, BD LSRFortessa SORP, SONY Spectral Cell Analyzer SP6800, BD LSRII flow sorter, BD FACSAria SORP flow sorter, SONY Sy3200 flow sorter
Software	For data collection we used SP6800 v1.6.3 and FACS Diva v6.1.<br>For data analysis we used FlowJo v10.4.2 or v10.4.0
Cell population abundance	We determined the abundance and morphology of post-sorted cell populations by manually counting in a Neubauer chamber.
Gating strategy	The gating strategies can be found in Supplementary Table 4.<br>Boundaries between "positive" and "negative" staining cells was determine by negative controls in populations that were clearly defined, by FMO in ki67+ and BrdU+ cell populations, and by the secondary and Isotype control antibodies for GATA-6+ cells withing ovary tumors.

- [x] Tick this box to confirm that a figure exemplifying the gating strategy is provided in the Supplementary Information.
